# Centella Asiatica Alleviates Type 2 Diabetes-Related Hepatic Glycolipid Disorders via Regulating UPP1-Mediated Pyrimidine Metabolism

**DOI:** 10.3390/cimb48070663

**Published:** 2026-06-27

**Authors:** Yunjiao Shen, Yuanyuan Yao, Zhihui Liu, Yi Li, Shijie Cao, Xinchi Feng

**Affiliations:** 1 School of Chinese Materia Medica, Tianjin Key Laboratory of Therapeutic Substance of Traditional Chinese Medicine, and China-Ghana Belt and Road Joint Laboratory on Traditional Medicine, Tianjin University of Traditional Chinese Medicine, Jinghai District, Tianjin 301617, China; syj795520@163.com (Y.S.); yaoyuanyuan01@163.com (Y.Y.); lzh521032025@163.com (Z.L.); 15369620697@163.com (Y.L.); 2 State Key Laboratory of Component-Based Chinese Medicine, Tianjin University of Traditional Chinese Medicine, Tianjin 301617, China

**Keywords:** *Centella asiatica*, asiatic acid, pyrimidine metabolism, uridine phosphorylase 1, uridine diphosphate glucose

## Abstract

Type 2 diabetes mellitus (T2DM) is a metabolic disorder characterized by glycolipid dysregulation and hepatic steatosis. Centella asiatica (CA) and its triterpenoid constituents exert metabolic benefits. In addition, previous metabolomics study found that asiatic acid regulated pyrimidine metabolism in obese mice, while the key target and pathway were undefined. This study investigated the regulatory effects of CA and its active constituents on T2DM-related glycolipid disorders, focusing on the pyrimidine metabolism pathway. T2DM mice were established using a high-fat diet combined with streptozotocin (STZ) and treated with Centella asiatica ethanolic extract or asiatic acid (AA), with glibenclamide as a positive control. Then, glycolipid metabolism, hepatic function, pyrimidine metabolites, and related mechanisms were assessed using biochemical assays, LC–MS/MS, cellular experiments, molecular analyses, and molecular docking. CAE and AA significantly reduced FBG (decreased by 51.01% and 53.01%), improved glucose intolerance, corrected dyslipidemia, alleviated hepatic steatosis, and attenuated insulin resistance in T2DM mice. They elevated hepatic uridine, cytidine, and UDP-glucose (UDPG) levels, promoted glycogen synthesis, inhibited uridine phosphorylase 1 (UPP1) activity, upregulated UDPG synthesis genes (PGM1, UGP2), and downregulated lipogenic genes (ACACA, Fasn, SREBP1/2). Molecular docking indicated specific binding of AA and asiaticoside to UPP1. This work distinguishes from our prior research by identifying UPP1 as a functional target and elucidating the detailed molecular mechanism. CA improves T2DM-associated glycolipid disorders and hepatic injury by modulating the pyrimidine metabolism-UDPG-glycogen synthesis pathway and targeting UPP1, highlighting its therapeutic potential for metabolic diseases.

## 1. Introduction

Carbohydrate and lipid metabolism are central physiological processes that regulate the body’s energy supply and material conversion. Imbalances in these processes are the primary causes of metabolic diseases such as fatty liver disease and type 2 diabetes mellitus (T2DM) [1]. T2DM is a chronic metabolic disorder with a high global prevalence. It is primarily characterized by persistent hyperglycemia, dyslipidemia, and insulin resistance. This condition often coexists with hepatic steatosis and impaired liver and kidney function, posing a serious threat to human health [2]. Although currently available clinical hypoglycemic and lipid-regulating medications can effectively manage symptoms to some extent, prolonged use may result in adverse effects, including hypoglycemia, gastrointestinal distress, and increased strain on hepatic and renal function [3]. Furthermore, these pharmaceutical agents have shown limited efficacy in reversing carbohydrate and lipid metabolism disorders and hepatic metabolic damage [3,4]. Therefore, the identification of safe, effective, and multi-targeted bioactive compounds of natural origin for T2DM intervention and treatment, along with its associated complications, has become a critically important research priority in the field of metabolic diseases [5,6,7].

Centella asiatica (CA) is a traditional medicinal and edible plant widely used in China, India, and many other regions of Asia. It serves not only as a medicinal herb but is also incorporated into vegetable dishes and beverages [8]. The primary active components of CA are pentacyclic triterpenoids, including asiatic acid (AA), madasiatic acid (MA), asiaticoside (AC), madecassic acid (MC), and asiaticoside B (AC-B) [9,10,11]. Modern pharmacological studies have indicated that CA and its triterpenoid components exhibit anti-inflammatory, antioxidant, insulin-sensitizing, and lipid-regulating properties, showing significant therapeutic potential in metabolic disease models such as obesity and diabetes [12,13,14,15,16]. Furthermore, extensive research has revealed its anti-steatotic and hepatoprotective effects [17]. For example, an animal model study using a high-fat diet induce hyperlipidemia showed that a crude ethanol extract of Centella asiatica can reduce serum triglyceride levels, enhance hepatic antioxidant capacity, and alleviate lipid accumulation in the liver [18]. Additionally, asiatica acid, a triterpenoid component, has been found to significantly inhibit de novo lipid synthesis in the liver by suppressing the sterol regulatory element-binding protein 1c (SREBP1c) signaling pathway, thereby improving insulin resistance induced by a high-fat diet [19]. Although our previous untargeted metabolomics study preliminarily indicated that asiatic acid regulates pyrimidine metabolism and alleviates metabolic disorders in ob/ob mice [20], the core functional targets, detailed molecular regulatory cascades, and mechanisms in clinically relevant type 2 diabetes models remain unclear. Thus, the specific molecular mechanisms by which CA improves glucose and lipid metabolism in T2DM have yet to be fully elucidated, and its key targets and core regulatory pathways require further investigation.

Pyrimidine metabolism is a critical pathway for maintaining not only nucleotide synthesis but also energy metabolism and hepatic homeostasis [21]. Uridine, a fundamental pyrimidine nucleoside, functions as an essential intermediary connecting nucleotide metabolism with hepatic glycolipid metabolism. Variations in uridine concentrations directly influence the synthesis of UDP-glucose (UDPG), which subsequently promotes hepatic glycogen synthesis, leading to a reduction in blood glucose levels [22]. Simultaneously, the accumulation of uridine-dependent UDPG inhibits hepatic lipogenesis and lipid accumulation. Moreover, dysregulated uridine metabolism has been shown to disrupt insulin signaling pathways in the liver, thereby aggravating insulin resistance in T2DM [23]. Among these compounds, uridine, cytidine, and their downstream metabolite UDPG constitute essential substrates for hepatic glycogen synthesis. Additionally, uridine phosphorylase 1 (UPP1) functions as a critical rate-limiting enzyme in uridine catabolism, thereby directly modulating the concentrations of pyrimidine metabolites [21,22,24]. Numerous preclinical investigations have substantiated that increased expression of UPP1 leads to a reduction in intracellular uridine levels, a decrease in UDPG-mediated inhibition of the lipogenic transcription factor SREBP1c, and a consequent intensification of lipid accumulation within hepatocytes [25,26]. Consequently, elevated levels of UPP1 have been observed in insulin-resistant mice subjected to a high-fat diet (HFD) as well as in obese individuals with diabetes, suggesting a pathogenic association between increased UPP1 activity and metabolic dysfunction [21]. Therefore, UPP1 represents a critical target for the treatment of type 2 diabetes, highlighting the necessity for additional research in this area. Benzylacyclouridine (BAU), a well-established selective inhibitor of UPP1, is commonly employed as a positive control in related pharmacological studies [27]. Recent studies have revealed a significant association between disruptions in pyrimidine metabolism and the aberrant activation of UPP1, which is linked to impaired glucose tolerance, increased lipid synthesis, and the development of hepatic steatosis in diabetic conditions [23]. Based on the results of previous metabolomics research, it has been proposed that AA may achieve its therapeutic effects through the regulation of pyrimidine metabolic pathways [20]. We propose that CA and its bioactive constituents exert antidiabetic effects through the modulation of UPP1, thereby influencing the pyrimidine metabolism-UDPG-glycogen synthesis pathway.

In this study, a mouse model of T2DM induced by a HFD and STZ, concomitant with hepatic steatosis, was established to investigate whether CA and its principal triterpenoid, asiatic acid, ameliorate glycolipid metabolic dysfunction through UPP1-related pyrimidine metabolism. Comprehensive biochemical and molecular analyses were conducted to validate this regulatory mechanism, elucidate the UPP1-mediated anti-steatotic effects of CA, and provide experimental evidence supporting its potential therapeutic application in metabolic disorders.

## 2. Materials and Methods

### 2.1. Materials and Reagents

Asiatic acid (AA, Cat#C11869220), Glibenclamide (INN, Cat#C10438568) and Anhydrous Glucose (Cat#C11821341) were obtained from Shanghai Macklin Biochemical Co., Ltd. (Shanghai, China). Madecassic acid (MA, (Cat#B20970), Asiaticoside (AC, Cat#B20586), Madecassoside (MC, Cat#B20969), Asiaticoside B (AC-B, Cat#B22023), Streptozocin (STZ, Cat#JS245968), Uridine (Cat#B20907), Uracil (Cat#B20908), Thymidine (Cat#B30633), Thymine (Cat#B25426) and Cytidine (Cat#B20073) were commercially supplied by Yuanye Bio-Technology Co., Ltd. (Shanghai, China). CAE was prepared and provided by our laboratory [28], its main components include AC (21.94%), MC (24.83%), AA (11.52%), MA (23.22%), AC-B (12.86%). The reagent kits utilized for the biochemical indicator assays, encompassing aspartate aminotransferase (AST), alanine aminotransferase (ALT), total cholesterol (TC), high-density lipoprotein cholesterol (HDL-C), triglycerides (TG), low-density lipoprotein cholesterol (LDL-C), creatinine (CRE), and mouse insulin enzyme-linked immunosorbent assay (ELISA) kits, were obtained from Nanjing Jiancheng Biotechnology Research Institute Co., Ltd. For the liquid chromatography-tandem mass spectrometry (LC–MS/MS) analyses, LC–MS-grade solvents including acetonitrile, methanol, formic acid, and water were employed.

### 2.2. Animal Experimental Design

**Figure 1:**
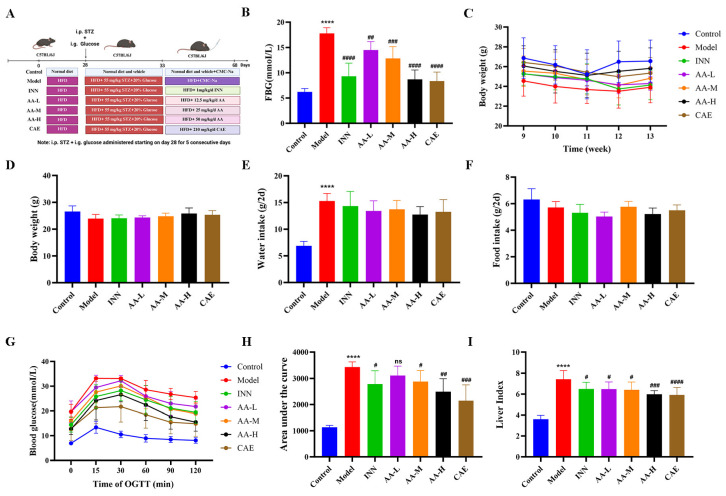
CA ameliorate glucose metabolism disorder and related metabolic parameters in T2DM mice. (**A**) Flowchart of the animal experimental protocol. (**B**) FBG levels in each group. (**C**) Body weight alterations throughout the intervention course. (**D**) Final body weight. (**E**) Water intake and (**F**) food intake per 2 days. (**G**) Oral glucose tolerance test (OGTT) curves. (**H**) AUC of OGTT. (**I**) Liver index. Values shown as the mean ± SEM (*n* = 8–10). **** *p* < 0.0001 compared with the Control group; # *p* < 0.05, ## *p* < 0.01, ### *p* < 0.001, #### *p* < 0.0001, ns stands for not significant compared with the Model group.

All experimental procedures involving mice were performed following the receipt of ethical approval from the Animal Experiment Ethics Committee of Tianjin University of Traditional Chinese Medicine (Approval No. TCM-LAEC2024159y3251). Female C57BL/6J mice, aged five weeks and weighing between 18 and 20 g, were procured from Beijing HFK Biotechnology Co., Ltd. (Beijing, China). The animals were maintained under standardized laboratory conditions, including a controlled temperature of 22 ± 1 °C, relative humidity of 50–60%, and a 12-h light–dark cycle. Throughout the study, mice had ad libitum access to standard laboratory chow and drinking water. Following a one-week acclimatization period, the mice were randomly assigned to two groups: a normal control group (*n* = 10) and a diabetic model group (*n* = 92). The animal modeling protocol and associated experimental procedures employed in this study were adapted and refined based on methodologies previously reported by Du et al. [29]. In the experimental design, mice in the model group were subjected to a HFD, whereas the control group received a standard chow diet. Following a four-week dietary intervention, the model group mice were intraperitoneally administered STZ dissolved in a 0.1 mg/L sodium citrate buffer (pH 4.5) at a dosage of 55 mg/kg/day for five consecutive days. Three days subsequent to the final STZ injection, mice exhibiting fasting blood glucose (FBG) levels exceeding 11.1 mmol/L after a 6-h fast were classified as having successfully developed a T2DM model. The 60 mice in which the model was successfully established were maintained on a high-fat diet and subsequently allocated to various experimental groups, including the Model, INN, AA-L, AA-M, AA-H, and CAE groups (*n* = 10 mice/ group). The experimental protocol is illustrated in Figure 1A. The INN, AA-L, AA-M, AA-H, and CAE groups received intragastric administrations of INN at 1 mg/kg/day, AA at 12.5 mg/kg/day, 25 mg/kg/day, and 50 mg/kg/day, and CAE at 210 mg/kg/day, respectively. The control and model groups were administered an equivalent volume of 0.5% sodium carboxymethylcellulose (CMC-Na) solution via intragastric gavage. Throughout the treatment period, daily food and water consumption for each mouse were recorded every two days, while body weight was measured weekly to monitor metabolic alterations induced by the high-fat diet and pharmacological interventions. At the conclusion of the experimental timeline, all mice underwent a 12-h fasting period, followed by anesthesia induction through exposure to 2% isoflurane aerosol. Blood samples were collected from the abdominal aorta and centrifuged at 3500× *g* for 8 min at 4 °C to separate serum from whole blood. Subsequently, mice were euthanized humanely by cervical dislocation, and their livers were excised, weighed, and sectioned. One portion of the liver tissue was allocated for histopathological examination, while the remaining samples were rapidly frozen in liquid nitrogen and stored at −80 °C for subsequent analyses.

### 2.3. Glucose Tolerance Test (GTT)

Serum insulin and fasting blood glucose levels were measured independently using a blood glucose monitor (Jiangsu Yuyue Medical Equipment & Supply Co., Ltd., Nanjing, China) and ELISA kits (Nanjing Jiancheng Biotechnology Research Institute Co., Ltd., Nanjing, China), respectively. The homeostatic model assessment of insulin resistance (HOMA-IR) index was calculated employing the standard formula: HOMA-IR = [fasting insulin (μU/mL) × fasting blood glucose (mmol/L)]/22.5. For the GTT, all mice underwent a 12-h overnight fast. Following baseline blood glucose measurement, each animal received a single intraperitoneal injection of glucose at a dose of 2 g/kg. Peripheral blood samples were collected from the tail vein at 15, 30, 60, 90, and 120 min post-glucose administration for subsequent blood glucose analysis [30].

### 2.4. Biochemistry Indexes

Serum TC, LDL-C, TG, AST, HDL-C, ALT, CRE, FINS, and ISI levels were determined with commercially supplied kits according to the provided protocols. For the determination of liver TG and TC, liver tissue was homogenized in absolute alcohol to prepare tissue extracts. For cell samples, the same detection kits were adopted; cell extracts were obtained by lysing cells with phosphate-buffered saline (PBS), followed by centrifugation to collect the supernatant for detection.

### 2.5. Hematoxylin and Eosin (H&E)

Liver tissues were fixed in 4% paraformaldehyde for at least 24 h, and subsequently embedded in paraffin and sliced into 5-μm sections. After pretreatment, the sections were subjected to hematoxylin and eosin. Histopathological changes of the liver were viewed and photographed under a light microscope (Nikon, Tokyo, Japan). Three non-adjacent sections from each mouse were selected for microscopic evaluation.

### 2.6. Oil Red O (ORO) Staining

Fresh liver tissues were prepared into frozen sections and stained with ORO working solution. After staining, the sections were washed with 60% isopropanol and distilled water to remove excess dye. Cell nuclei were counterstained with hematoxylin, and histological lipid deposition was observed under an Olympus BX60 light microscope (Olympus (China) Co., Ltd., Beijing, China). Three non-adjacent sections from each mouse were analyzed to guarantee reliable and representative results. Lipid accumulation was quantified via ImageJ (1.50i) analysis of Oil Red O staining intensity.

### 2.7. Preparation of Standard Solution

Accurately weighed uridine, uracil, cytidine, thymidine and the internal standard thiamazole were fully suspended in DMSO to formulate a stock solution at 10 mg/mL. These stock solutions were further made up with 80% methanol to configure the mixed working solution. In parallel, thiamazole serving as the internal standard was diluted in 80% methanol to yield a 100 ng/mL internal standard working liquid.

### 2.8. Preparation of the Liver Sample

Liver tissue (50 mg) was accurately harvested from mice in each group and mixed with 80% methanol to extract metabolites. Tissue homogenization was performed using a high-speed homogenizer (SWE-FP, Wuhan Saivell Biotechnology Co., Ltd., Wuhan, China). The liver homogenate was spun at 8000 rpm for 10 min under 4 °C conditions, and the upper liquid phase was recovered for subsequent analysis. Afterwards, 100 μL of tissue supernatant was admixed with 20 μL internal standard solution. After vigorous shaking for 2 min, the mixture was centrifuged at 14,000 rpm for 10 min. Transfer the final supernatant to a chromatography tube in preparation for subsequent instrumental analysis.

### 2.9. LC–MS/MS Conditions

Detection was conducted with a Waters UPLC–MS/MS platform (UPLC I-Class Xevo TQ-XS, Waters, Milford, MA, USA), employing an ACQUITY UPLC HSS T3 column (2.1 mm × 100 mm, 1.8 μm). The mobile phases consisted of 0.1% formic acid aqueous solution (A) and high-performance liquid chromatography-grade acetonitrile (B). The flow rate was maintained 0.3 mL/min with a gradient elution schedule: 0–1 min, 2–10% B; 1–2.5 min, 10–60% B; 2.5–3 min, 60–2% B; 3–5 min, maintained at 2% B. An injection volume of 2 µL was used, with the column oven maintained at 10 °C.

Mass spectrometry analysis was completed with a Waters Xevo TQ-XS triple quadrupole mass spectrometer. Electrospray ionization (ESI) in positive mode with multiple reaction monitoring (MRM) was adopted for QqQ–MS data acquisition. Key mass spectral parameters were configured as follows: ion source temperature, 120 °C; desolvation gas temperature, 400 °C; desolvation gas flow, 800 L/h; nebulizer pressure, 7.0 bar; capillary voltage, 3.0 kV; and cone voltage, 2 V. The MRM transition parameters for all target metabolites were fully optimized to ensure accurate quantification (Table S1).

### 2.10. UDPG Content Determination

Liver sample pretreatment and mass spectrometry parameters remained consistent with the above-described procedures. Chromatographic separation was implemented on a Waters UPLC I-Class Xevo TQ-XS system (Waters, Milford, MA, USA) fitted with an ACQUITY UPLC HSS T3 column (2.1 mm × 100 mm, 1.8 μm). The mobile phase contained 5 mM ammonium acetate containing 0.1% formic acid (A) and acetonitrile (B). The gradient elution schedule was set as: 0–1.5 min, 95% B; 1.5–2.0 min, 95–80% B; 2.0–2.5 min, 80–95% B; 2.5–3.5 min, sustained at 95% B. The injection volume was 2 μL, and the column temperature was maintained at 10 °C.

### 2.11. Glycogen Content Assay

Liver glycogen content was determined using a commercial glycogen assay kit (Suzhou Grace Biotechnology Co., Ltd., Suzhou, China, Cat. No. G0590W96). Accurately weighed liver samples (20 mg) from each group were mixed with 160 μL glycogen extraction buffer and ultrasonically lysed to obtain tissue homogenate. The homogenate was divided into testing and blank control subgroups. The experimental subgroup received 55 μL of distilled water, whereas the control subgroup was supplemented with 80 μL of pure water. All samples were incubated at 95 °C for 3 min, followed sequentially by natural brought down to room temperature, followed by the addition of amyloglucosidase. The mixtures were incubated at 37 °C for 1.5 h to ensure complete hydrolysis of hepatic glycogen into glucose, then subjected to centrifugation at 12,000 rpm for 5 min to collect the supernatants. Briefly, 40 μL supernatant, 10 μL glucose oxidase reagent and 140 μL chromogenic solution were sequentially loaded into a 96-well plate. The samples were gently mixed and then kept in the dark at room temperature for 20 min. The optical density at 510 nm was finally detected to calculate hepatic glycogen content.

### 2.12. DHODH Enzymatic Assay

Stock solutions of AA, MA, AC, MC, AC-B, L-dihydroorotic acid, and positive control brequinar (Brq) were diluted with assay buffer to 2 mM (L-dihydroorotic acid) and 4 μM (test compounds and Brq). Reactions were performed in 96-well plates at 37 °C. Each well contained 50 μL substrate-free mixture, 25 μL test compound or Brq, and was incubated for 10 min. Reactions were initiated by adding 25 μL L-dihydroorotic acid (final concentration 500 μM). Absorbance at 600 nm was recorded kinetically every 10 s for 8 min [31]. Blank (no substrate) and negative (equal volume of solvent) controls were included. Inhibition was calculated as:

$$\mathrm{Inhibition}\;\mathrm{rate}\;(\%)\;=\;[1\;-\;(V_{\mathrm{compound}}\;-\;V_{\mathrm{blank}})/(V_{\mathrm{control}}\;-\;V_{\mathrm{blank}})]\;\times \;100\%$$

V_(compound)_, V_(blank)_, and V_(control)_ represent initial velocities of the test, blank, and control groups, respectively.

### 2.13. UPP1 Enzymatic Assay

Stock solutions of CA active components, positive control BAU, and substrate uridine were serially diluted with 0.1 M potassium phosphate buffer (pH 7.4) to 40 μM (test compounds), 4 μM (BAU), and 200 μM (uridine), respectively. The microplate reader was preheated to 37 °C. In a 96-well UV plate, 50 μL of UPP1 protein (2 ng/μL) was pipetted into each well, followed by 25 μL of test compound or BAU solution; the control group received an equal volume of DMSO. After 1 min incubation at 37 °C, the reaction was triggered by the addition of 25 μL uridine (final concentration 50 μM). Kinetic absorbance was recorded at 280 nm every 5 s for 5 min [32,33]. The inhibition rate was calculated based on initial reaction velocity:

$$\mathrm{Inhibition}\;\mathrm{rate}\;(\%)\;=\;(1\;-\;V_{\mathrm{compound}}/V_{\mathrm{control}})\;\times \;100\%$$

V_compound_ and V_control_ represent the initial velocities of the treated and control groups, respectively.

### 2.14. Cell Culture and Treatment

HepG2 cell line was supplied by Wuhan Procell Life Science & Technology Co., Ltd. (catalog No. CL-0103). The HepG2 cells were regularly incubated in DMEM medium fortified with 10% fetal bovine serum and kept at 37 °C in an incubator with 5% CO_2_ atmosphere. Referring to cell activity and intracellular triglyceride levels, 0.4 mM oleic acid was applied to construct an in vitro lipid overaccumulation model. After successful modeling, cells were intervened with different agents for 24 h, including fenofibrate (10 μM), UDPG (100 μM), CAE (10 μg/mL), as well as equal concentrations (10 μM) of AA, MA, AC, MC and AC-B.

### 2.15. Boron-Dipyrromethene (BODIPY) Staining

HepG2 cells were seeded in 96-well opaque plates with transparent bottoms, stained with PBS containing 5 μmol/L BODIPY and Hoechst 33342, and incubated for 30 min. Subsequently, the fluorescent signal intensity within individual cells was quantified using high-throughput imaging analysis (Cytiva, NY, USA).

### 2.16. Quantitative Real-Time PCR (qRT-PCR)

Total RNA was attributed to HepG2 cells in each group using TRIzol reagent (Tiangen Biotech Co., Ltd., Beijing, China). RT-qPCR analysis were conducted with the ABI 7500 real-time PCR instrument. The relative transcriptional levels of the target genes were scaled using β-actin as a constitutive control and quantified using the 2^−△△CT^ method.

### 2.17. Immunohistochemical Staining

Liver samples from each group were fixed with 4% paraformaldehyde, embedded in paraffin, and cut into serial sections of 5 μm thickness. The sections were then dewaxed in xylene and rehydrated through graded ethanol solutions. The samples were rinsed with 3% hydrogen peroxide for 25 min at room temperature in the dark, followed by antigen retrieval using PBS with a pH of 7.4. All sections were sealed with 10% goat serum for 1 h, followed by overnight incubation at 4 °C with primary antibodies targeting UPP1 (Servicebio, Wuhan, China, Cat. GB11532, 1:200) and DHODH (Servicebio, Wuhan, China, GB113375, 1:500). Afterwards, the sections were maintained in HRP-labeled secondary antibodies. Positive signals were visualized using the DAB staining method, and cell nuclei were counterstained with hematoxylin. Finally, histological images were acquired using a Nikon ECLIPSE E100 bright-field microscope (Tokyo, Japan). Immunohistochemical staining was quantified using the H-score method.

### 2.18. Molecular Docking

Semi-flexible docking analyses were carried out via the AutoDock Vina program (version 4.2.6), with UPP1 as the receptor and AA, MA, AC, MC, and AC-B as ligands. The crystal structure of UPP1 (PDB ID: 3EUE) was accessed from the RCSB protein data bank, while ligand structures were obtained from PubChem (CIDs: 119034, 73412, 11954171, 24825675, 91618002). Protein preparation (dehydration and ligand removal) was conducted using PyMOL (version 2.6.2). AutoDockTools (MGLTools package) was used for hydrogenation, charge calculation, and grid box generation. Visualization and interaction analysis were implemented using PyMOL (2.6.2) and Discovery Studio software (2022).

### 2.19. Statistical Analysis

All quantitative analyses were performed using GraphPad Prism version 9.5. The Shapiro–Wilk test and Levene’s test were employed to assess the assumptions of normality and homogeneity of variances, respectively, with all datasets satisfying the requirements for parametric statistical methods. One-way analysis of variance (ANOVA) was utilized for comparisons across groups, followed by Tukey’s Honestly Significant Difference (HSD) test for pairwise post hoc comparisons; this procedure inherently corrects for multiple comparison errors. Sample sizes for each experimental assay are explicitly indicated in the figure legends and the main text. Potential outliers were evaluated using Grubbs’ test at a significance level of α = 0.05; no outliers or missing data points were detected in any of the datasets. Data are expressed as the mean ± standard deviation (SD), and statistical significance was defined as *p* < 0.05.

## 3. Result

### 3.1. CA Alleviates Hyperglycemia and Improves Glucose Metabolism in T2DM Mice

According to the experimental protocol illustrated in Figure 1A, the therapeutic potential of CA in mitigating HFD-induced T2DM was evaluated in a murine model. Monitoring of FBG levels revealed that mice in the model group consistently exhibited FBG concentrations exceeding 11.1 mmol/L, significantly elevated compared to the control group, thereby confirming the successful establishment of a stable diabetic model. Administration of INN, as well as graded doses of AA and CAE, resulted in a substantial reduction in circulating FBG levels in diabetic mice (Figure 1B and Table S2). No significant differences in body weight were observed among the experimental groups throughout the intervention period (Figure 1C,D). Regarding water and food intake, excessive water consumption was specifically observed in the HFD-fed model mice (*p* < 0.0001), whereas interventions with AA and CAE did not significantly affect either food or water consumption (Figure 1E,F). To investigate whether AA and CAE could ameliorate glucose metabolic dysfunction under conditions of lipid metabolic disturbance, a glucose tolerance test was conducted following four consecutive weeks of treatment. Dynamic blood glucose profiles were generated, and the corresponding AUC was calculated for quantitative assessment. The model group demonstrated a significantly elevated AUC compared to normal controls (*p* < 0.0001), indicative of pronounced glucose intolerance induced by HFD. Conversely, treatment with INN, as well as medium and high doses of AA and CAE, significantly decreased the AUC values, suggesting an improvement in glucose clearance capacity. However, a significant reduction in AUC levels was not detected in the AA-L group (Figure 1G,H). Analysis of the liver index revealed pronounced hepatomegaly in the model mice, as indicated by markedly elevated liver index values compared to the control group. This pathological liver enlargement was substantially ameliorated following treatment with INN, AA at varying dosages, and CAE, as evidenced by significant decreases in liver index across all intervention groups (*p* < 0.05; Figure 1I).

### 3.2. CA Alleviates Dyslipidemia, Organ Injury and Insulin Resistance in T2DM Mice

**Figure 2:**
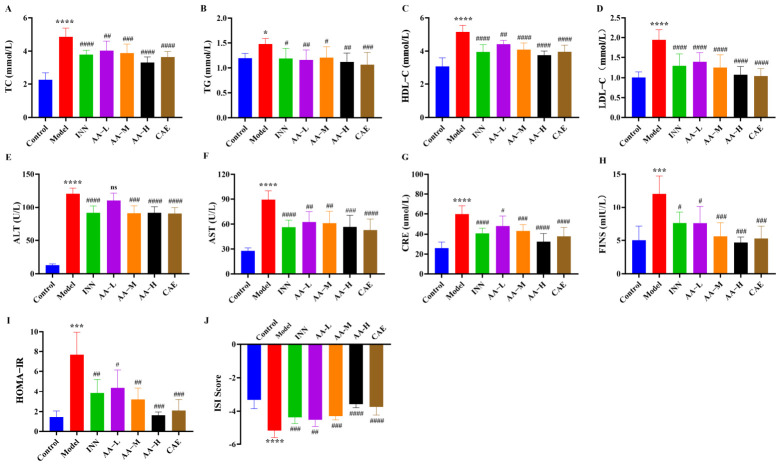
Effects of CA on circulating lipid levels, insulin resistance indices and hepatic and renal biochemica function in T2DM mice. The levels of (**A**) TC, (**B**) TG, (**C**) HDL-C, (**D**) LDL-C, (**E**) ALT, (**F**) AST, (**G**) CRE, (**H**) FLNS, (**I**) HOMA-IR and (**J**) ISI score. Data represent mean ± SD (*n* = 6). Statistical significance: #### *p* < 0.0001, ### *p* < 0.001, ## *p* < 0.01, # *p* < 0.05 compared with the Model group. **** *p* < 0.0001, *** *p* < 0.001, * *p* < 0.05 compared with the Control group.

To further investigate the modulatory effects of AA and CAE on disordered glucose and lipid metabolism, key serum biochemical parameters were measured across all experimental groups. Compared to the control group, the model mice exhibited significantly elevated serum levels of HDL-C, LDL-C, TC, and TG (*p* < 0.0001), indicating pronounced dyslipidemia in the diabetic mice. In contrast, treatment with INN, as well as low, medium, and high doses of AA (AA-L, AA-M, AA-H) and CAE, significantly reduced these lipid parameters in the model animals (Figure 2A–D). To evaluate hepatic injury in the T2DM mice, serum levels of ALT, AST, and CRE were assessed. The model group demonstrated markedly elevated concentrations of these liver injury markers, whereas administration of INN, various doses of AA, and CAE effectively reversed these abnormal increases (Figure 2E–G). Furthermore, circulating insulin levels were significantly increased in the model group relative to the normal control group (*p* < 0.001), while treatment with INN, AA, and CAE notably suppressed insulin secretion (*p* < 0.05 or *p* < 0.001). Concurrently, interventions with AA and CAE substantially ameliorated insulin resistance and restored insulin sensitivity in diabetic mice (Figure 2H–J). Collectively, these results indicate that both AA and CAE alleviate disturbances in glycolipid metabolism and hepatic dysfunction. Moreover, these treatments exert beneficial effects on insulin metabolic disorders by reducing insulin resistance and enhancing insulin responsiveness.

### 3.3. CA Ameliorates Hepatic Steatosis and Liver Damage in T2DM Mice

**Figure 3:**
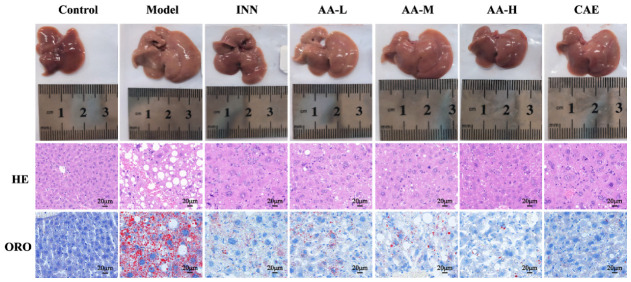
Effects of CA on hepatic gross morphology, histopathological changes and lipid deposition in T2DM mice. The upper panel shows the gross morphology of mouse livers in each group (n = 10). The middle panel presents HE staining of liver tissues (n = 3, scale bar = 20 μm), and the lower panel shows ORO staining for hepatic lipid droplets (*n* = 3, scale bar = 20 μm).

H&E staining, along with ORO staining, were employed to assess histopathological alterations and lipid accumulation in liver tissues across various treatment groups. As depicted in Figure 3 and Figure S1, liver specimens from the model group exhibited a substantial presence of lipid vacuoles and extensive red-stained lipid droplets compared to the normal control group. Consistent with the effects observed in the positive control group treated with INN, administration of AA and CAE significantly inhibited excessive hepatic lipid accumulation. Collectively, these findings indicate that both AA and CAE confer notable hepatoprotective effects by reducing lipid deposition and ameliorating liver tissue damage.

### 3.4. CA Increases the Levels of Endogenous Pyrimidine Components in Hepatic Tissues of T2DM Mice

**Figure 4:**
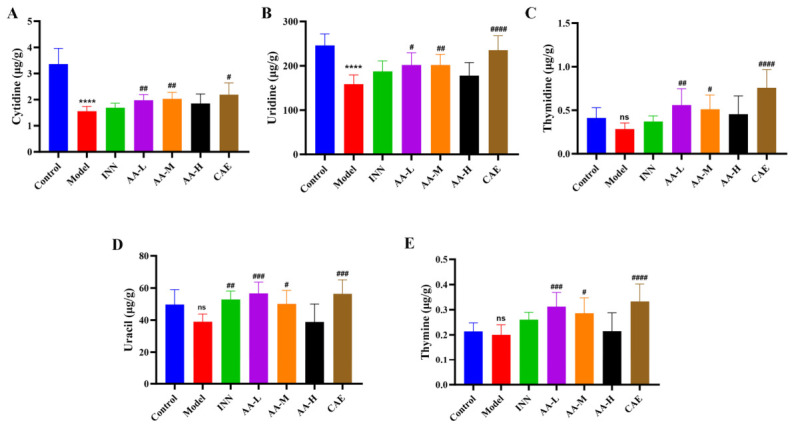
Effects of CA on the levels of endogenous pyrimidine metabolites in the liver of mice (*n* = 10). (**A**) Uridine content; (**B**) Uracil content; (**C**) Cytidine content; (**D**) Thymidine content; (**E**) Thymine content. Data are expressed as the mean ± standard deviation (SD), (**** *p* < 0.0001 compared with the control group; # *p* < 0.05, ## *p* < 0.01, ### *p* < 0.001, #### *p* < 0.0001, ns stands for not significant compared with the model group.).

It has been reported in prior investigations that AA could mitigate metabolic disorders in ob/ob mice by adjusting pyrimidine metabolism [20]. This study investigated alterations in pyrimidine concentrations across different animal groups following administration of AA and CAE. Notably, LC–MSMS was employed to develop a quantitative analytical method targeting five endogenous pyrimidine compounds—uridine, uracil, thymidine, thymine, and cytidine—in mouse liver tissue. The method was rigorously validated, with validation data provided in Supplementary Figure S2 and Tables S3–S7. Utilizing this validated quantitative approach, the concentrations of the five pyrimidine components in mouse liver samples were quantified. The findings revealed a significant reduction in uridine and cytidine levels in the livers of model group mice compared to controls (*p* < 0.0001). The concentrations of uracil, thymidine, and thymine did not demonstrate significant changes. However, relative to the model group, the AA-L, AA-M, and CAE groups showed a statistically significant increase in the levels of cytidine, uridine, uracil, thymidine, and thymine within liver tissue (*p* < 0.05). The AA-H group had no significant effect. The most pronounced effects following drug intervention were observed in the levels of uridine, uracil, and cytidine (Figure 4, Table S8).

### 3.5. CA Promotes Hepatic Glycogen Synthesis by Upregulating UDPG Levels and Rectifying Pyrimidine Metabolism in T2DM Mice

**Figure 5:**
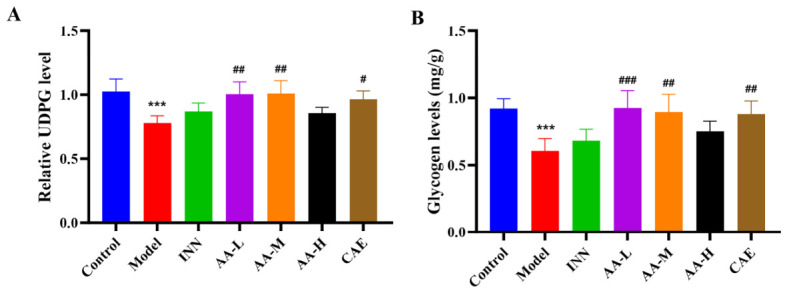
Effects of CA on hepatic UDPG levels and glycogen synthesis in diabetic mice. (**A**) Relative UDPG levels in liver tissues. (**B**) Hepatic glycogen content. Data are presented as the mean ± SEM (*n* = 5). *** *p* < 0.001 compared with the Control group; # *p* < 0.05, ## *p* < 0.01, ### *p* < 0.001 compared with the Model group.

Uridine is a pivotal precursor of UDPG, a direct substrate for glycogen synthesis [21]. In order to further investigate the mechanisms underlying CA’s hypoglycemic and lipid-regulating effects, the levels of liver glycogen and UDPG were examined in vivo. As demonstrated in Figure 5, in comparison with blank controls, hepatic UDPG and glycogen levels were notably lowered in the model group (*p* < 0.001). Following administration with AA-L and CAE, significant increases in UDPG and glycogen levels were observed (*p* < 0.05). Compared with the model group, the AA-L and CAE groups increased by 53.19% and 45.74%, respectively. However, administration of AA-H did not significantly affect UDPG or glycogen levels. The present study’s findings corroborate that CAE and AA can significantly upregulate UDPG levels in the mouse liver and promote glycogen synthesis.

### 3.6. The Effect of CA on Cellular Glycogen and Lipid Levels

**Figure 6:**
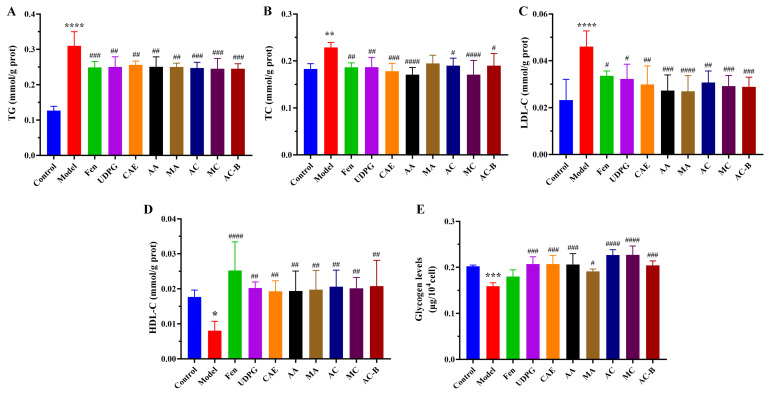
Alterations in glycogen and lipid levels of HepG2 cells. (**A**) TG content; (**B**) TC content; (**C**) LDL-C content; (**D**) HDL-C content; (**E**) Glycogen content. Data are expressed as the mean ± SD, *n* = 6. * *p* < 0.05, ** *p* < 0.01, *** *p* < 0.001, **** *p* < 0.0001 vs. the control group; # *p* < 0.05, ## *p* < 0.01, ### *p* < 0.001, #### *p* < 0.0001 compared with the model group.

Consequently, to further investigate the lipid-lowering effects of the major triterpenoid components in CA, we measured the relevant lipid levels at the cellular level. Fen is the standard positive control in experiments designed to assess the efficacy of agents that reduce serum lipids. Additionally, elevated UDPG levels have been shown to promote glycogen synthesis and inhibit lipogenesis. Therefore, it will be administered in subsequent experiments to establish a control group for the CA triterpenes in comparison with Fen and UDPG. This will facilitate a more thorough investigation of the lipid-lowering effects of these triterpenes. In addition, the dosages of OA, fen, and other drugs were validated in preliminary experiments to ensure they are non-toxic to cells (Figure S3). As revealed in Figure 6, relative to the control group, the model group showed markedly elevated TG, TC, and LDL-C and markedly decreased HDL-C and glycogen (*p* < 0.05). Treatment with UDPG, fenofibrate, AA, MA, AC, MC, AC-B, and CAE markedly decreased serum TG, TC, and LDL-C contents, alongside an obvious increase in HDL-C and hepatic glycogen contents (*p* < 0.05). Collectively, these results reveal that CA supplementation facilitates glycogen production and inhibits excessive lipogenesis.

### 3.7. The Effect of the Main Component of CA on Cellular Lipid Accumulation

**Figure 7:**
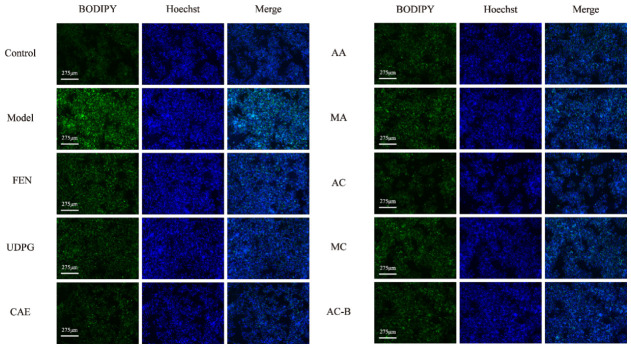
Effects of CA extract and its active components on intracellular lipid accumulation detected by BODIPY staining. (*n* = 6, microscope: 10×). Representative fluorescence images of BODIPY (green, lipid droplets) and Hoechst 33342 (blue, nuclei) staining in HepG2 cells. Scale bar = 275 μm.

To further investigate the effects of drug administration at the cellular level, BODIPY staining was employed to quantify lipid droplet accumulation. The results, presented in Figure 7, indicate that the model group exhibited a marked increase in lipid droplet accumulation compared to the blank control group. Treatment with the principal active compound of CA resulted in a significant reduction in lipid droplet number, producing effects comparable to those observed in the group treated with exogenous UDPG. These findings imply that the main active constituent of CA may mitigate lipid accumulation through the elevation of UDPG levels.

### 3.8. Regulation of Lipogenic and UDPG-Synthetic Gene Expression by CA Triterpenoids and UDPG in HepG2 Cells

**Figure 8:**
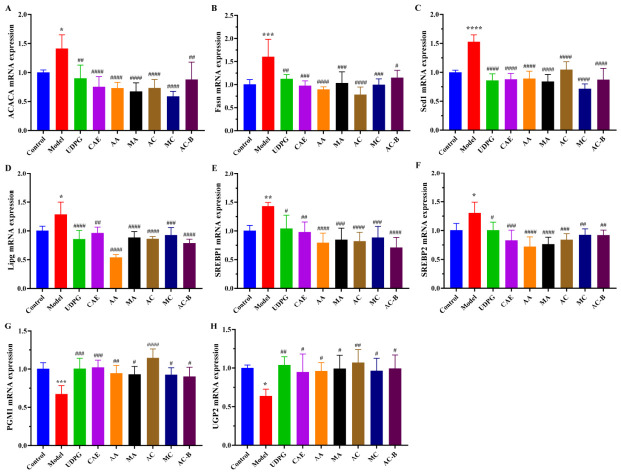
Effects of centella triterpenoids and UDPG on the mRNA expression abundance of lipogenesis-related genes and UDPG biosynthetic genes in HepG2 cells. (**A**–**F**) The mRNA expression levels of lipogenesis-related genes, including ACACA (**A**), Fasn (**B**), Scd1 (**C**), Lipg (**D**), SREBP1 (**E**), and SREBP2 (**F**); The mRNA expression levels of UDPG biosynthetic genes PGM1 (**G**) and UGP2 (**H**). Data are presented as the mean ± SD, *n* = 5. * *p* < 0.05, ** *p* < 0.01, *** *p* < 0.001, **** *p* < 0.0001 compared with the control group; # *p* < 0.05, ## *p* < 0.01, ### *p* < 0.001, #### *p* < 0.0001 compared with the model group.

Lipid metabolism is tightly modulated through expression levels of genes relevant to lipid synthesis and catabolism. Accordingly, the mRNA levels of lipogenesis-related genes, including ACACA, Fasn, Scd1, Lipg, SREBP1, and SREBP2, were determined in HepG2 cells using RT-qPCR. All amplification primers were designed and synthesized by Beijing Dingguo Changsheng Biotechnology Co., Ltd. (Beijing, China). (Table S9). As illustrated in Figure 8A–F, all lipogenic genes presented markedly elevated expression in the model group as compared to the control group. Exposure to UDPG, centella triterpenoids, or CAE markedly reversed these elevations. Furthermore, the expression of genes concerned with UDPG biosynthesis was also examined. As depicted in Figure 8G,H, the mRNA levels of PGM1 and UGP2 were notably reduced in the model group, whereas UDPG, triterpenoid components, and CAE treatment markedly reversed these decreases. Altogether, these findings demonstrate that CA effectively enhances the expression of UDPG biosynthetic genes while suppressing the transcription of key lipogenic genes.

### 3.9. The Effect of CA on UPP1 Enzyme Activity

**Figure 9:**
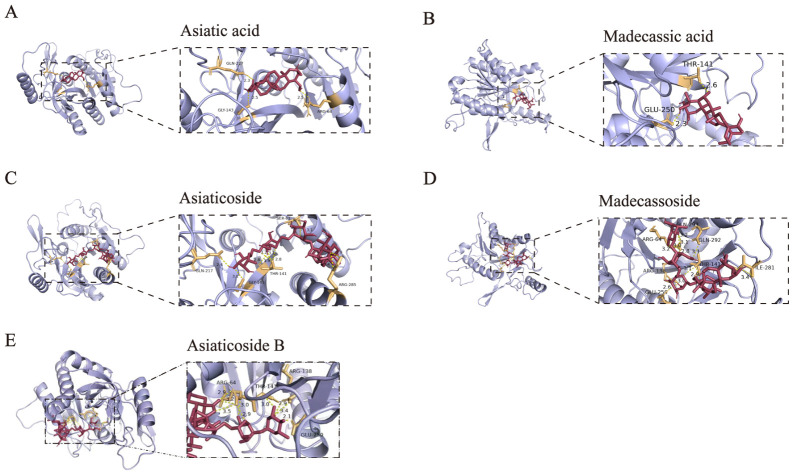
Molecular docking models of five triterpenoid compounds from CA with target protein. (**A**–**E**) The 3D binding conformations and detailed interactions between the target protein and five triterpenoid compounds: (**A**) AA, (**B**) MA, (**C**) AC, (**D**) MC, and (**E**) AC-B.

The previously mentioned research findings suggest that the hypoglycemic and lipid-regulating effects of CA are closely associated with its enhancement of pyrimidine metabolism and modulation of UDPG levels. Consequently, immunohistochemical analysis was conducted to examine the impact of AA and CAE on the expression of DHODH and UPP1. The results indicated that treatment with AA and CAE did not significantly affect the protein expression levels of either enzyme (Figure S4). Subsequently, we conducted a series of enzyme activity tests, the results of which indicated that AA, MA, AC, MC, and AC-B had no effect on DHODH activity (Figure S5). In contrast, AA, MA, AC, MC, and AC-B all inhibited UPP1 activity, with inhibition rates of 71%, 61%, 73%, 72%, and 77%, respectively (Table 1). This finding indicates that the triterpenoid components of CA may elicit their hypoglycemic and lipid-regulating effects by inhibiting UPP1. Finally, molecular docking was utilized to clarify the binding affinity between Centella asiatica triterpenoids and UPP1. The results demonstrated that AA, MA, AC, MC, and AC-B exhibited potential binding affinity for UPP1 (Figure 9 and Table S10), thereby providing further evidence to support the hypothesis that Centella asiatica triterpenoids may alleviate metabolic dysfunction partially via suppressing UPP1 activity.

## 4. Discussion

CA is a significant medicinal plant that has been utilized in various regions of the world, including the East, and is currently experiencing a surge in popularity in the West [34]. In addition to its conventional medicinal applications, CA has also been extensively utilized in the food and cosmetics industries [35]. CA has been shown to possess antioxidant, anti-inflammatory, wound-healing, anxiolytic, antibacterial, anti-tumor, and neuroprotective properties [36]. Consequently, CA is frequently employed to address diabetic complications, Alzheimer’s disease, and to enhance wound healing in diabetic patients [37,38]. Accumulated evidence has indicated that CA exerts beneficial effects on modulating glucose and lipid metabolism in diabetic rats [13]. The present study thus constructed a diabetic mouse model via a high-sugar and high-fat diet combined with STZ injection, and administered different concentrations of AA and CAE for treatment. The results demonstrated that these treatments could enhance lipid levels and glucose tolerance, reduce improve insulin sensitivity, and insulin resistance. Moreover, CAE and different concentrations of AA significantly improved hepatic morphology and histopathological abnormalities. These findings are consistent with those of previous studies, which collectively indicate that CA and their representative triterpenoid component, asiatic acid, can exert a crucial effect on diabetes intervention [13,39,40].

As demonstrated in our group’s earlier research, AA, a constituent triterpenoid of CA, has been observed to significantly enhance glucose and lipid metabolism in ob/ob mice, augment insulin sensitivity, and reduce lipid accumulation [20]. A comprehensive analysis encompassing non-targeted liver metabolomics has identified the regulation of the pyrimidine metabolic pathway as a pivotal mechanism underlying these effects. Pyrimidine metabolism is a network mediated by specific enzymes, in which various components are interconnected and maintain a dynamic equilibrium. Uridine and uracil are reversibly interconverted by UPP1 in the hepatic pyrimidine salvage pathway. Cytidine deaminase drives the unidirectional transformation of cytidine into uridine, while thymidylate synthase acts solely on de novo thymidine production and is not involved in pyrimidine nucleoside cycling. Uric acid, a terminal purine metabolite, cannot be mutually converted with uridine through UPP1 or other pyrimidine-processing enzymes [41]; Cytidine can be directly converted to uridine through deamination; uridine is closely linked to thymidine metabolism via deoxygenation and methylation processes [42]. The present study therefore established an LC–MS/MS method for the parallel measurement of the levels of five major endogenous pyrimidines in liver tissue. In addition, the study examined changes in the levels of these five pyrimidine components in liver tissue following administration of AA and CAE. The results demonstrated that AA-L, AA-M, and CAE led to a significant increase in uridine and cytidine levels in the liver, while uracil, thymidine, and thymidylate also increased to a certain extent. Among these, the CAE group demonstrated greater efficacy, likely due to its diverse active components, which, in addition to AA, include other triterpenoids such as MA, AC, MC, and AC-B. Furthermore, the presence of flavonoids, organic acids, and other substances has been documented [28]. Consequently, subsequent experiments validated the bioactive roles of other components. Of particular significance is the observation that while the levels of uridine and cytidine both underwent significant changes, uridine serves as the biosynthetic precursor of cytidine. Indeed, uridine drives cytidine production via the UCK2-UTP-CTP pathway [43]. Based on this metabolic pathway, it is hypothesized that the primary regulatory target of AA is the uridine metabolism pathway, with the observed downregulation of cytidine levels representing a downstream secondary consequence of uridine modulation. Multiple studies have demonstrated that AA can activate the AMPK/SIRT1 energy-sensing axis, restore Akt/GSK-3β insulin signaling, inhibit the TNF-α/NF-κB inflammatory cascade, enhance Nrf2-mediated antioxidant defenses, and suppress the expression of lipid synthesis-related proteins such as SREBP1c, ACC1, and FASN. Collectively, these mechanisms contribute to the mitigation of hepatic steatosis [19]. Furthermore, additional research has shown that AA protects pancreatic β-cell function through the TNF-α/Mfn2 signaling pathway, thereby supporting the preservation of normal insulin secretion [44]. Consequently, the alleviation of hyperglycemia, impaired glucose tolerance, dyslipidemia, and insulin resistance observed in our animal and cellular models stems from synergistic multi-target effects.

Glycogen synthesis and lipogenesis represent two cellular pathways for energy storage, and these two processes are antagonistic [26]. The injection of uridine diphosphate (UDP) has been shown to upregulate the levels of UDP-glucose (UDPG), a substrate for glycogen synthesis, thereby activating glycogen synthesis [24]. Concurrently, UDPG targets S1P in the Golgi apparatus, thereby inhibiting S1P-mediated cleavage of SREBPs. This, in turn, suppresses lipid synthesis in hepatocytes and promotes the storage of glucose as glycogen. Administration of UDPG to mouse models effectively treats metabolic dysfunction-associated steatohepatitis (MASLD) [26]. Furthermore, studies have demonstrated that GP2 functions as the rate-limiting enzyme in UDPG synthesis, and that UDPG can negatively allosterically regulate GP2 activity, thereby establishing a negative feedback loop. In this loop, UDPG regulates its own production, thus influencing the supply of precursors for glycogen and glycolipid synthesis [45]. The present study measured UDPG and glycogen levels in mouse liver tissue and found that AA-L and AA-M and CAE administration significantly increased UDPG and glycogen levels. This finding suggests that CA may also exert its lipid metabolism-regulating effects by increasing uridine levels.

As a human hepatocellular carcinoma cell line, HepG2 cells exhibit active pyrimidine metabolism and high levels of UPP1 expression [46,47]. Conversely, HepG2 cells maintain the lipid metabolic pathways characteristic of normal liver cells, thereby serving as a well-established model for investigating lipid metabolism disorders implicated in non-alcoholic fatty liver disease and type 2 diabetes. This renders them particularly well-suited for the purpose of lipid metabolism investigations in this study [48,49]. The present study employed the HepG2 lipid accumulation model to investigate alterations in intracellular uridine, UDPG, and glycogen levels subsequent to the administration of the primary active components of CA. The findings indicated that the administration of active triterpenoid components from CA brought about a substantial downregulation in lipid levels, a reduction in lipid accumulation, and a downregulation in the expression levels of genes and transcription factors linked to lipid synthesis (ACACA, Fasn, Scd1, Lipg, SREBP1, and SREBP2). Concurrently, there was a rise in the expression levels of genes correlated with UDPG synthesis (PGM1 and UGP2).

Furthermore, uridine levels can be regulated at multiple pivotal points in uridine synthesis and metabolism, thereby modulating glucose homeostasis and lipid metabolism [22]. With respect to the process of synthesis, DHODH is situated within the inner mitochondrial membrane and functions as the key regulatory enzyme in the de novo pyrimidine synthesis pathway [50]. It promotes the pivotal reaction of dehydrogenating dihydroorotic acid to form orotic acid. This step directly influences the synthesis of the uridine precursor UMP [51]. Inhibition of DHODH activity has been shown to result in a decrease in uridine levels [52] in mice and patients, concurrently promoting glycolysis, mitigating pancreatic β-cell damage, and improving glucose metabolism disorders in diabetic mice [53]. It is important to highlight that our in vitro enzymatic assays demonstrated that CA triterpenoids did not exhibit any measurable inhibition of DHODH activity. This negative finding reinforces the specificity of the UPP1-centered regulatory mechanism we have proposed. Numerous synthetic pyrimidine inhibitors, along with various plant-derived polyphenols, have been reported to concurrently inhibit both enzymes [54]. Such dual inhibition has been associated with extensive disruption of the intracellular pyrimidine pool, thereby complicating the elucidation of the precise underlying mechanisms [55]. These observations further support the antidiabetic potential of CA triterpenoids, which appears to be mediated through the selective inhibition of UPP1-dependent uridine degradation. This mode of action is distinct from the interference with DHODH-driven de novo pyrimidine biosynthesis reported in other investigations [56]. With regard to metabolism, UPP1, as the rate-limiting enzyme in uridine catabolism, has been demonstrated to determine the rate of uridine breakdown [57]. A significant reduction in uric acid levels was observed in the plasma and liver tissue of UPP1-knockout mice, accompanied by severe hepatic lipid accumulation. The administration of uric acid through dietary supplementation effectively ameliorated the observed lipid accumulation in UPP1-knockout mice [23]. This study investigated the effects of CA on two critical enzymes involved in uridine synthesis and metabolism. Immunohistochemical analysis of liver tissue demonstrated that both centellic acid and the CAE did not significantly alter the protein expression levels of DHODH and UPP1. Further examination revealed that the principal active compound of CA does not affect DHODH enzymatic activity but significantly inhibits UPP1 activity. Moreover, molecular docking analyses suggest that this key active constituent of CA possesses a potential binding affinity for UPP1. These findings imply that CAE may mitigate metabolic dysfunction, at least in part, through the suppression of UPP1 activity.

The present study identified the pyrimidine metabolic pathway on which CA relies in order to exert its hypoglycemic and lipid-regulating effects. However, it is important to note that there are several limitations that should be taken into account in order to guide future research. The investigation utilized only HFD/STZ-induced diabetic mice and HepG2 hepatoma cells as experimental models, both of which inadequately replicate the complex pathophysiology of human T2DM and the physiology of primary hepatocytes. The association between UPP1 inhibition and enhanced hepatic metabolism remains correlational; thus, genetic or pharmacological rescue experiments are necessary to establish causality. Several mechanistic findings are preliminary: enzyme inhibition assays were conducted at a single concentration without dose–response characterization or determination of half-maximal inhibitory concentration (IC50) values; gene expression analyses were limited to mRNA levels without corresponding protein quantification; molecular docking studies lacked quantitative binding parameters such as binding energies and identification of critical interacting residues. Metabolite quantification relied on relative peak intensities rather than absolute calibrated concentrations, and statistical reporting was restricted to *p*-values without inclusion of effect sizes or confidence intervals, limiting assessment of biological relevance.

Furthermore, data on in vivo organ toxicity, pharmacokinetics, and translational applicability of CAE and AA are currently insufficient. Given that CA exhibits broad anti-inflammatory, antioxidant, and insulin-sensitizing properties, its metabolic benefits cannot be solely attributed to UPP1-mediated pyrimidine remodeling. The observed superior efficacy of the whole CA extract compared to isolated AA suggests potential synergistic interactions among its phytochemical constituents, which warrants further detailed investigation.

## 5. Conclusions

Overall, the present study corroborates the hypothesis that CAE and AA effectively alleviate the following symptoms in mice with type 2 diabetes: hyperglycemia, dyslipidemia, hepatic steatosis, and insulin resistance. The observed beneficial effects are achieved by inhibiting UPP1 activity, improving pyrimidine metabolism, increasing hepatic UDPG and glycogen levels, and suppressing excessive lipid synthesis. Taken together, these findings underscore the metabolic regulatory function of CA in alleviating type 2 diabetes and identify UPP1 as a potential target for further mechanistic studies. This research provides a solid foundation for the development of CA as a potential natural medicine for the treatment of type 2 diabetes and related metabolic diseases.

## Figures and Tables

**Table 1:** Inhibition rate of UPP1 activity in vitro (*n* = 5).

Compound	UPP1 Inhibition Rate (%)
BAU	86
Asiatic acid	71
Madecassic acid	61
Asiaticoside	73
Madecassoside	72
Asiaticoside B	77

## Data Availability

The datasets analyzed during the current study are available from the corresponding authors upon reasonable request.

## Supplementary Materials

The following supporting information can be downloaded at: https://www.mdpi.com/article/10.3390/cimb48070663/s1.

## References

[1] Ferdous S.E., Ferrell J.M. (2024). Pathophysiological Relationship between Type 2 Diabetes Mellitus and Metabolic Dysfunction-Associated Steatotic Liver Disease: Novel Therapeutic Approaches. Int. J. Mol. Sci..

[2] GBD 2021 Diabetes Collaborators (2023). Global, regional, and national burden of diabetes from 1990 to 2021, with projections of prevalence to 2050: A systematic analysis for the Global Burden of Disease Study 2021. Lancet.

[3] Młynarska E., Czarnik W., Dzieża N., Jędraszak W., Majchrowicz G., Prusinowski F., Stabrawa M., Rysz J., Franczyk B. (2025). Type 2 Diabetes Mellitus: New Pathogenetic Mechanisms, Treatment and the Most Important Complications. Int. J. Mol. Sci..

[4] Ciccarelli G., Di Giuseppe G., Cinti F., Moffa S., Mezza T., Giaccari A. (2023). Why do some glucose-lowering agents improve non-alcoholic fatty liver disease whereas others do not? A narrative review in search of a unifying hypothesis. Diabetes Metab. Res. Rev..

[5] Sun B., Wu L., Wu Y., Zhang C., Qin L., Hayashi M., Kudo M., Gao M., Liu T. (2020). Therapeutic Potential of *Centella asiatica* and Its Triterpenes: A Review. Front. Pharmacol..

[6] Chahrour J.A., Abdel Baki Z., El Badan D., Nasser G., Maresca M., Hijazi A. (2025). Herbal Medicines in the Management of Diabetes Mellitus: Plants, Bioactive Compounds, and Mechanisms of Action. Biomolecules.

[7] Zhao Y.L., Liao J.B., Pang P.P., Li J.Y., Su S.C., Shao M.Q., Wen W.B., Xu F.R. (2025). Pharmacological advances in multi-targeted strategies for type 2 diabetes mellitus: A systematic perspective based on traditional Chinese medicine. Front. Pharmacol..

[8] Lim J., Lee H., Hong S., Lee J., Kim Y. (2024). Comparison of the Antioxidant Potency of Four Triterpenes of *Centella asiatica* against Oxidative Stress. Antioxidants.

[9] Zhou P., Yang P., Zhang K., Guo H., Du J., Huang L., Jin D., Alolga R.N., Wang H., Li J. (2025). Discovery and engineering of the asiaticoside, madecassoside and asiaticoside B biosynthetic pathway. Plant Physiol. Biochem..

[10] Liang C., Ma Y., Ding M., Gao F., Yu K., Wang S., Qu Y., Hua H., Li D. (2025). Asiatic acid and its derivatives: Pharmacological insights and applications. Eur. J. Med. Chem..

[11] Li W., Xu K., Lan M., Gao J., Dou L., Yang Y., Wang Q., Yan M., Li S., Ma Q. (2024). The dual functions of the pentacyclic triterpenoid madecassic acid in ameliorating doxorubicin-induced cardiotoxicity and enhancing the antitumor efficacy of doxorubicin. Int. J. Biol. Sci..

[12] Barinda A.J., Arozal W., Dwita N.C., Safutra M.S., Shimizu I., Hsiao Y.T., Sandora N., Hakim R.W., Khatimah N.G., Hardi H. (2025). *Centella asiatica* extract improves senescence-associated metabolic dysfunction by targeting inflammation in adipose tissue and macrophage in obesity-induced insulin resistance mice. Front. Endocrinol..

[13] Maulidiani, Abas F., Khatib A., Perumal V., Suppaiah V., Ismail A., Hamid M., Shaari K., Lajis N.H. (2016). Metabolic alteration in obese diabetes rats upon treatment with *Centella asiatica* extract. J. Ethnopharmacol..

[14] Uddandrao V.V.S., Rameshreddy P., Brahmanaidu P., Ponnusamy P., Balakrishnan S., Ramavat R.N., Swapna K., Pothani S., Nemani H., Meriga B. (2020). Antiobesity efficacy of asiatic acid: Down-regulation of adipogenic and inflammatory processes in high fat diet induced obese rats. Arch. Physiol. Biochem..

[15] Pakdeechote P., Bunbupha S., Kukongviriyapan U., Prachaney P., Khrisanapant W., Kukongviriyapan V. (2014). Asiatic acid alleviates hemodynamic and metabolic alterations via restoring eNOS/iNOS expression, oxidative stress, and inflammation in diet-induced metabolic syndrome rats. Nutrients.

[16] Rameshreddy P., Uddandrao V.V.S., Brahmanaidu P., Vadivukkarasi S., Ravindarnaik R., Suresh P., Swapna K., Kalaivani A., Parvathi P., Tamilmani P. (2018). Obesity-alleviating potential of asiatic acid and its effects on ACC1, UCP2, and CPT1 mRNA expression in high fat diet-induced obese Sprague-Dawley rats. Mol. Cell. Biochem..

[17] Zhao Y., Shu P., Zhang Y., Lin L., Zhou H., Xu Z., Suo D., Xie A., Jin X. (2014). Effect of *Centella asiatica* on oxidative stress and lipid metabolism in hyperlipidemic animal models. Oxid. Med. Cell. Longev..

[18] Kumari S., Deori M., Elancheran R., Kotoky J., Devi R. (2016). In vitro and In vivo Antioxidant, Anti-hyperlipidemic Properties and Chemical Characterization of *Centella asiatica* (L.) Extract. Front. Pharmacol..

[19] Yan S.L., Yang H.T., Lee Y.J., Lin C.C., Chang M.H., Yin M.C. (2014). Asiatic acid ameliorates hepatic lipid accumulation and insulin resistance in mice consuming a high-fat diet. J. Agric. Food Chem..

[20] Niu K., Bai P., Yang B., Feng X., Qiu F. (2022). Asiatic acid alleviates metabolism disorders in ob/ob mice: Mechanistic insights. Food Funct..

[21] Yang Y., Ye Y., Deng Y., Gao L. (2024). Uridine and its role in metabolic diseases, tumors, and neurodegenerative diseases. Front. Physiol..

[22] Zhang Y., Guo S., Xie C., Fang J. (2020). Uridine Metabolism and Its Role in Glucose, Lipid, and Amino Acid Homeostasis. Biomed. Res. Int..

[23] Urasaki Y., Pizzorno G., Le T.T. (2016). Chronic Uridine Administration Induces Fatty Liver and Pre-Diabetic Conditions in Mice. PLoS ONE.

[24] Urasaki Y., Pizzorno G., Le T.T. (2014). Uridine affects liver protein glycosylation, insulin signaling, and heme biosynthesis. PLoS ONE.

[25] Le T.T., Ziemba A., Urasaki Y., Hayes E., Brotman S., Pizzorno G. (2013). Disruption of uridine homeostasis links liver pyrimidine metabolism to lipid accumulation. J. Lipid Res..

[26] Chen J., Zhou Y., Liu Z., Lu Y., Jiang Y., Cao K., Zhou N., Wang D., Zhang C., Zhou N. (2024). Hepatic glycogenesis antagonizes lipogenesis by blocking S1P via UDPG. Science.

[27] Niedzwicki J.G., Chu S.H., el Kouni M.H., Rowe E.C., Cha S. (1982). 5-benzylacyclouridine and 5-benzyloxybenzylacyclouridine, potent inhibitors of uridine phosphorylase. Biochem. Pharmacol..

[28] Jin X., Zang J., Li A., Yao Y., Dai Z., Feng X., Qiu F. (2026). Analysis of the Chemical Composition and Glycolipid Metabolism-Regulating Properties of *Centella asiatica*. Chem. Biodivers..

[29] Wu S.X., Zhao X.Y., Yang Y.H., Zhou M.Q., Zheng Y., Wu Z.J., Zou Q.Y., Zhang T.T., Du L. (2025). Krill oil alleviates type 2 diabetes mellitus-induced sarcopenia in mice via attenuating insulin resistance, intestinal barrier dysfunction, and skeletal muscle protein turnover impairment. Food Funct..

[30] Chen M., Liu G., Fang Z., Gao W., Song Y., Lei L., Du X., Li X. (2025). Buddleoside alleviates nonalcoholic steatohepatitis by targeting the AMPK-TFEB signaling pathway. Autophagy.

[31] Khairy A., Hammoda H.M., Celik I., Zaatout H.H., Ibrahim R.S. (2022). Discovery of potential natural dihydroorotate dehydrogenase inhibitors and their synergism with brequinar via integrated molecular docking, dynamic simulations and in vitro approach. Sci. Rep..

[32] Renck D., Machado P., Souto A.A., Rosado L.A., Erig T., Campos M.M., Farias C.B., Roesler R., Timmers L.F., de Souza O.N. (2013). Design of novel potent inhibitors of human uridine phosphorylase-1: Synthesis, inhibition studies, thermodynamics, and in vitro influence on 5-fluorouracil cytotoxicity. J. Med. Chem..

[33] Renck D., Ducati R.G., Palma M.S., Santos D.S., Basso L.A. (2010). The kinetic mechanism of human uridine phosphorylase 1: Towards the development of enzyme inhibitors for cancer chemotherapy. Arch. Biochem. Biophys..

[34] Oyenihi A.B., Chegou N.N., Oguntibeju O.O., Masola B. (2017). *Centella asiatica* enhances hepatic antioxidant status and regulates hepatic inflammatory cytokines in type 2 diabetic rats. Pharm. Biol..

[35] Zeng W., Li H., Liu S., Luo Z., Chen J., Zhou J. (2025). Biosynthesis and bioactivities of triterpenoids from *Centella asiatica*: Challenges and opportunities. Biotechnol. Adv..

[36] Jiang J., Han R., Ren H., Yao Y., Jiang W. (2025). A review of neuroprotective properties of *Centella asiatica* (L.) Urb. and its therapeutic effects. Ann. Med..

[37] Pattanaik S.K., Mohanty D., Ray A., Jena S., Pattanaik S., Rath D. (2026). Phytotherapy for ageing-related multimorbidity: Systems-level insights into *Centella asiatica* in diabetes and Alzheimer’s Disease. Phytomedicine.

[38] Dağar O., Ortatatlı M. (2026). Effects of *Centella asiatica* extract on burn wound healing in streptozotocin-induced diabetic rats: A pathological and molecular evaluation. J. Mol. Histol..

[39] Oyenihi A.B., Langa S.O.P., Mukaratirwa S., Masola B. (2019). Effects of *Centella asiatica* on skeletal muscle structure and key enzymes of glucose and glycogen metabolism in type 2 diabetic rats. Biomed. Pharmacother..

[40] Ramachandran V., Saravanan R. (2013). Efficacy of asiatic acid, a pentacyclic triterpene on attenuating the key enzymes activities of carbohydrate metabolism in streptozotocin-induced diabetic rats. Phytomedicine.

[41] Wang C.C., Cheng H.W. (1984). Salvage of pyrimidine nucleosides by *Trichomonas vaginalis*. Mol. Biochem. Parasitol..

[42] Xia J.F., Wang Z.H., Liang Q.L., Wang Y.M., Li P., Luo G.A. (2011). Correlations of six related pyrimidine metabolites and diabetic retinopathy in Chinese type 2 diabetic patients. Clin. Chim. Acta.

[43] Fu Y., Wei X.D., Guo L., Wu K., Le J., Ma Y., Kong X., Tong Y., Wu H. (2022). The Metabolic and Non-Metabolic Roles of UCK2 in Tumor Progression. Front Oncol..

[44] Li L., Wang W., Xu Q., Huang M. (2023). Asiatic acid improves insulin secretion of β cells in type 2 diabetes through TNF-α/Mfn2 pathway. J. Zhejiang Univ. (Med. Sci.).

[45] Decker D., Kleczkowski L.A. (2018). UDP-Sugar Producing Pyrophosphorylases: Distinct and Essential Enzymes with Overlapping Substrate Specificities, Providing de novo Precursors for Glycosylation Reactions. Front. Plant Sci..

[46] Okumura M., Iwakiri T., Yoshikawa N., Nagatomo T., Ayabe T., Tsuneyoshi I., Ikeda R. (2022). Hepatocyte Growth Factor Enhances Antineoplastic Effect of 5-Fluorouracil by Increasing UPP1 Expression in HepG2 Cells. Int. J. Mol. Sci..

[47] da Silva E.F.G., Lima K.G., Krause G.C., Haute G.V., Pedrazza L., Catarina A.V., Gassen R.B., de Souza Basso B., Dias H.B., Luft C. (2020). CPBMF65, a synthetic human uridine phosphorylase-1 inhibitor, reduces HepG2 cell proliferation through cell cycle arrest and senescence. Investig. New Drugs.

[48] Gu Y., Duan S., Ding M., Zheng Q., Fan G., Li X., Li Y., Liu C., Sun R., Liu R. (2022). Saikosaponin D attenuates metabolic associated fatty liver disease by coordinately tuning PPARα and INSIG/SREBP1c pathway. Phytomedicine.

[49] Li X., Ge J., Li Y., Cai Y., Zheng Q., Huang N., Gu Y., Han Q., Li Y., Sun R. (2021). Integrative lipidomic and transcriptomic study unravels the therapeutic effects of saikosaponins A and D on non-alcoholic fatty liver disease. Acta Pharm. Sin. B.

[50] Luganini A., Boschi D., Lolli M.L., Gribaudo G. (2025). DHODH inhibitors: What will it take to get them into the clinic as antivirals?. Antivir. Res..

[51] Yang C., Zhao Y., Wang L., Guo Z., Ma L., Yang R., Wu Y., Li X., Niu J., Chu Q. (2023). De novo pyrimidine biosynthetic complexes support cancer cell proliferation and ferroptosis defence. Nat. Cell Biol..

[52] Peters G.J., Schwartsmann G., Nadal J.C., Laurensse E.J., van Groeningen C.J., van der Vijgh W.J., Pinedo H.M. (1990). In vivo inhibition of the pyrimidine de novo enzyme dihydroorotic acid dehydrogenase by brequinar sodium (DUP-785; NSC 368390) in mice and patients. Cancer Res..

[53] Zhang J., Terán G., Popa M., Madapura H., Ladds M., Lianoudaki D., Grünler J., Arsenian-Henriksson M., McCormack E., Rottenberg M.E. (2021). DHODH inhibition modulates glucose metabolism and circulating GDF15, and improves metabolic balance. iScience.

[54] Skinner O.S., Blanco-Fernández J., Goodman R.P., Kawakami A., Shen H., Kemény L.V., Joesch-Cohen L., Rees M.G., Roth J.A., Fisher D.E. (2023). Salvage of ribose from uridine or RNA supports glycolysis in nutrient-limited conditions. Nat. Metab..

[55] Walter M., Herr P. (2022). Re-Discovery of Pyrimidine Salvage as Target in Cancer Therapy. Cells.

[56] Izzo A.A., Teixeira M., Alexander S.P.H., Cirino G., Docherty J.R., George C.H., Insel P.A., Ji Y., Kendall D.A., Panattieri R.A. (2020). A practical guide for transparent reporting of research on natural products in the British Journal of Pharmacology: Reproducibility of natural product research. Br. J. Pharmacol..

[57] Ward M.H., Nwosu Z.C., Lyssiotis C.A. (2023). Uridine: As sweet as sugar for some cells?. Cell Res..

