# Supplementary material for: Centella Asiatica Alleviates Type 2 Diabetes-Related Hepatic Glycolipid Disorders via Regulating UPP1-Mediated Pyrimidine Metabolism
Source: Curr Issues Mol Biol. 2026 Jun 27;48(7):663. doi: 10.3390/cimb48070663 (PMC13406324; doi:10.3390/cimb48070663)
Supplement: Supplementary file 1 [file cimb-48-00663-s001.zip › cimb-4381343-supplementary.pdf]

Supplementary Information

Table S1. Mass scan method parameters of MRM

Compound	Precursor Ion (m/z)	Product Ion (m/z)	Cone Voltage (V)	Collision Energy (eV)
Uridine	245.17	113.10	18	8
Uracil	113.11	43.06	24	16
Cytidine	244.18	112.06	18	10
Thymidine	243.19	127.11	18	16
Thymine	127.18	54.11	12	20
IS	115.13	57.06	10	14

Table S2. Comparison of FBG in each group before and after administration（n = 10）

Group	Number (units)	Before treatment（mmol/l）	After treatment（mmol/l）
Control	10	7.6 ± 0.6	6.2 ± 0.7
Model	10	22.2 ± 1.9	17.9 ± 1.3
INN	10	20.3 ± 2.4	9.3 ± 2.6
AA-L	10	22.6 ± 2.3	14.5 ± 1.7
AA-M	10	21 ± 1.9	12.8 ± 2.3
AA-H	10	19.9 ± 2.2	8.7 ± 1.8
CAE	10	20.5 ± 3.3	8.4 ± 1.8

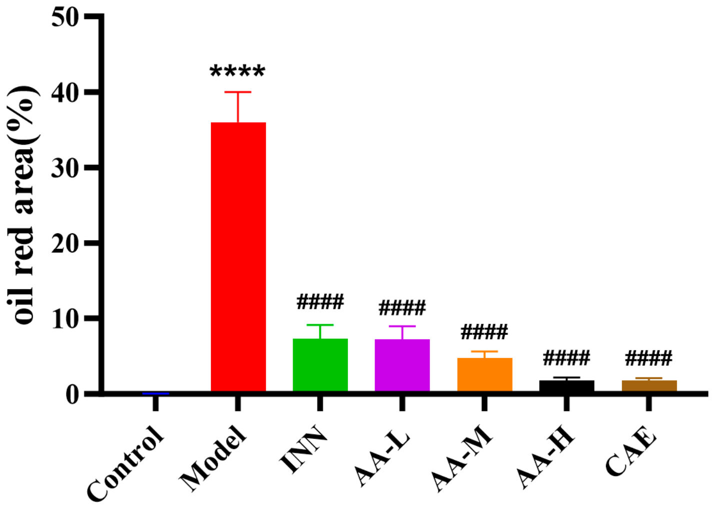

Figure S1. Quantitative Oil Red O Staining of Mouse Liver Tissue.Compared with the Control group, ****$P < 0.0001$; compared with the Model group, ####$P < 0.0001$, $n = 3$

S1. Examination of exclusivity

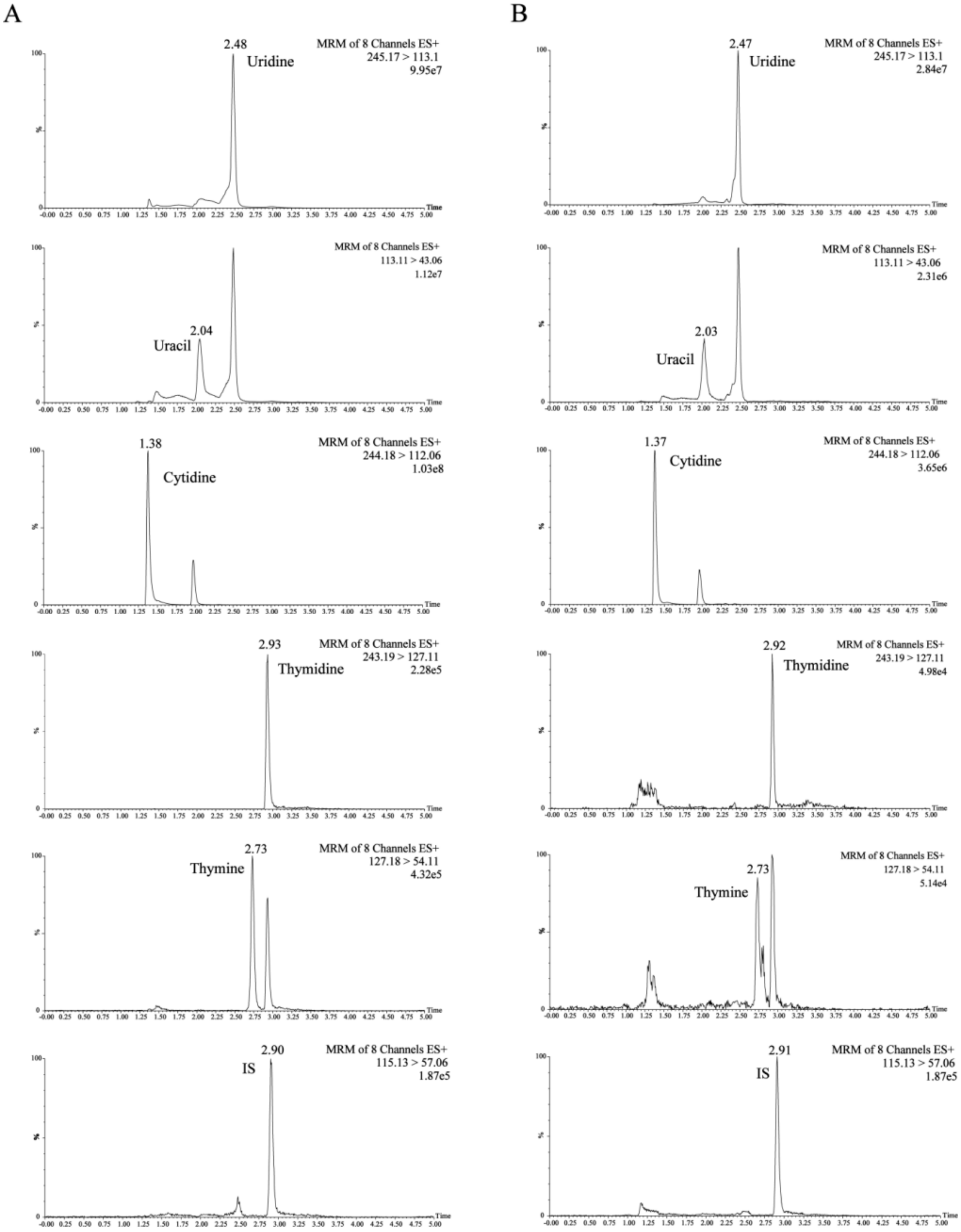

Figure S2. Specificity chromatogram of each pyrimidine component. (A) Chromatographic diagram of each pyrimidine component standard; (B) Chromatogram of pyrimidine components in liver samples.

Table S3. Linear equations, correlation coefficients, linear ranges and lower limits of quantitation of the five pyrimidine components

Compound	Calibration curves	Linear range （ng·mL^{-1}）	r	LLOQ （ng·mL^{-1}）	RE （%）	RSD （%）
Uridine	Y=0.00456391X-16.8382	10000.0-125000	0.9985	10000.0	8.1	1.6
Uracil	Y=0.0012588X-0.273553	1000.0-40000	0.9978	1000.0	5.1	4.0
Cytidine	Y=0.117904X-7.9599	100.0-4000	0.9987	100.0	11.8	2.4
Thymidine	Y=0.00128285X-0.00184114	5.00-500	0.9997	5.00	7.3	7.9
Thymine	Y=0.00730786X-0.015601	5.00-400	0.9990	5.00	9.0	10.7

Precision and accuracy

Table S4. Intra-day and inter-day precision and accuracy of five pyrimidine components

Compound	Spiked Conc. (ng·mL^{-1})	Intra-day (n = 6)			Inter-day (n = 18)		
		Measured Conc. (ng·mL^{-1})	Accuracy (%)	RSD (%)	Measured Conc. (ng·mL^{-1})	Accuracy (%)	RSD (%)
Uridine	20000.0	20401.5 ± 196.0	102.0	1.0	20484.6 ± 630.0	102.4	3.1
	40000.0	40313.1 ± 151.5	100.8	0.4	40312.5 ± 943.6	100.8	2.3
	100000	103004 ± 1046.0	103.0	1.0	103865 ± 1526.2	103.9	1.5
Uracil	2000.0	1759.0 ± 98.4	88.0	5.6	1669.0 ± 114.2	83.5	6.8
	8000.0	8423.6 ± 381.6	105.3	4.5	7477.3 ± 825.8	93.5	11.0
	32000	31633 ± 341.3	98.9	1.1	31270 ± 1218.4	97.7	3.9
Cytidine	200.0	207.8 ± 2.4	103.9	1.2	218.3 ± 10.2	109.2	4.7
	800.0	804.8 ± 8.4	100.6	1.0	771.0 ± 35.0	96.4	4.5
	3200	3211 ± 79.2	100.4	2.5	3642 ± 361.6	113.8	9.9

Thymidine	10.0	10.8 ± 1.0	107.8	9.1	10.7 ± 0.9	107.4	8.1
	50.0	47.9 ± 2.3	95.7	4.8	48.5 ± 2.7	97.0	5.5
	400	408 ± 6.1	102.1	1.5	420 ± 19.6	105.0	4.7
Thymine	10.0	9.61 ± 0.4	95.7	4.6	10.7 ± 1.2	106.6	11.5
	50.0	53.0 ± 1.9	106.0	3.6	51.9 ± 1.9	103.8	3.6
	320	327 ± 2.4	102.1	0.7	345 ± 15.9	107.9	4.6

Spike recovery

Table S5. Recovery of five pyrimidines（n = 6）

	initial quantity (ng)	Marked quantity (ng)	measured quantity (ng)	Recover（%）	Average Recover（%）	RSD (%)
Uridine	1303.15	1296.36	2537.79	95.2	99.0	3.2
	1211.56	1296.36	2540.00	102.5		
	1390.77	1296.36	2699.41	100.9		
	1335.30	1296.36	2606.60	98.1		
	1281.63	1296.36	2517.92	95.4		
	1257.23	1296.36	2575.82	101.7		
Uracil	456.44	474.26	904.83	94.5	96.8	3.9
	442.64	474.26	889.72	94.3		
	451.58	474.26	896.31	93.8		
	443.12	474.26	899.50	96.2		
	496.13	474.26	988.77	103.9		
	477.94	474.26	943.28	98.1		
Cytidine	13.49	13.24	26.16	95.7	99.6	3.9
	12.39	13.24	26.22	104.5		
	14.91	13.24	28.29	101.1		
	15.21	13.24	28.35	99.3		

	13.00	13.24	26.54	102.3		
	13.13	13.24	25.63	94.5		
Thymidine	3.78	2.89	6.60	97.9	101.7	3.6
	3.10	2.89	6.14	105.4		
	2.66	2.89	5.48	97.7		
	2.20	2.89	5.18	103.3		
	3.60	2.89	6.48	99.8		
	2.30	2.89	5.36	106.1		
Thymine	0.87	1.18	2.11	105.1	98.8	3.9
	0.85	1.18	2.04	100.8		
	0.84	1.18	1.99	97.5		
	1.42	1.18	2.55	96.2		
	1.43	1.18	2.60	99.2		
	1.47	1.18	2.58	94.1		

Stability of the reference standard solution

Table S6. Stability of five pyrimidine components ($n = 6$)

Compound	Content	Spiked Conc. (ng·mL^{-1})	Measured Conc. (ng·mL^{-1})	RSD (%)
Uridine	4 h at room temperate	20000.0	20055.2 ± 218.7	1.1
		40000.0	40348.7 ± 268.2	0.7
		100000	102791 ± 1282.0	1.2
	24 h at auto-sampler	20000.0	20727.3 ± 655.6	3.2
		40000.0	40450.0 ± 346.3	0.9
		100000	107105 ± 855.2	0.8
	Storge at -80°C 7 days	20000.0	19698.8 ± 405.1	2.1
		40000.0	39776.9 ± 714.4	1.8
		100000	113094 ± 1116.1	1.0
Uracil	4 h at room temperate	2000.0	2045.9 ± 100.1	4.9

		8000.0	7722.2 ± 269.4	3.5
		32000	33075 ± 856.5	2.6
		2000.0	2322.3 ± 61.5	2.6
	24 h at auto-sampler	8000.0	8281.5 ± 313.2	3.8
		32000	32881 ± 303.8	0.9
		2000.0	1563.5 ± 47.8	3.1
	Storge at -80°C 7 days	8000.0	7952.4 ± 94.5	1.2
		32000	32645 ± 797.2	2.4
		200.0	198.5 ± 7.5	3.8
	4 h at room temperate	800.0	777.7 ± 16.9	2.2
		3200	3121 ± 135.7	4.3
		200.0	202.7 ± 3.0	1.5
Cytidine	24 h at auto-sampler	800.0	724.1 ± 23.4	3.2
		3200	3803 ± 110.1	2.9
		200.0	212.4 ± 11.2	5.3
	Storge at -80°C 7 days	800.0	752.5 ± 14.5	1.9
		3200	3952 ± 104.6	2.6
		10.0	9.82 ± 1.0	10.4
	4 h at room temperate	50.0	49.4 ± 2.9	5.9
Thymidine		400	450 ± 16.6	3.7
		10.0	9.13 ± 0.4	4.6
	24 h at auto-sampler	50.0	44.9 ± 0.9	1.9
		400	410 ± 18.1	4.4
		10.0	10.4 ± 1.3	12.1
Thymidine	Storge at -80°C 7 days	50.0	49.4 ± 2.1	4.3
		400	474 ± 30.2	6.4
		10.0	9.64 ± 0.8	8.3
Thymine	4 h at room temperate	50.0	48.9 ± 1.2	2.5
		320	353 ± 17.0	4.8

		10.0	9.04 ± 0.4	4.8
	24 h at auto-sampler	50.0	48.2 ± 2.9	6.0
		320	345 ± 12.7	3.7
		10.0	10.8 ± 1.0	9.0
	Storge at -80°C 7 days	50.0	50.1 ± 1.5	2.9
		320	385 ± 11.4	3.0

Stability of liver samples

Table S7. Sample stability of five pyrimidines (*n* = 6)

Compound	Content	Measured Conc. (ng/mL)	RSD(%)	RE(%)
Uridine	0 h at auto-sampler	16803.5 ± 914.1	5.4	-
	4 h at auto-sampler	16751.3 ± 1192.7	7.1	-0.3
	12 h at auto-sampler	15215.2 ± 2056.2	13.5	-9.5
	24 h at auto-sampler	15754.6 ± 879.9	5.6	-6.2
	Storage at -20℃ 1 days	15949.8 ± 920.6	5.8	-5.1
	2 Freeze-thaw cycles	15468.7 ± 1539.1	9.9	-7.9
	Storage at -80℃ 15 days	17513.6 ± 1261.3	7.2	4.2
	Storage at -80℃ 2 months	11411.1 ± 1491.0	13.1	-32.1
Uracil	0 h at auto-sampler	6842.8 ± 320.1	4.7	-
	4 h at auto-sampler	6625.1 ± 896.8	13.5	-3.2
	12 h at auto-sampler	6667.8 ± 913.7	13.7	-2.6
	24 h at auto-sampler	6463.9 ± 608.2	9.4	-5.5
	Storage at -20℃ 1 days	7047.6 ± 1021.3	14.5	3.0
	2 Freeze-thaw cycles	6218.3 ± 545.2	8.8	-9.1
	Storage at -80℃ 15 days	6240.5 ± 781.1	12.5	-8.8
	Storage at -80℃ 2 months	5035.3 ± 504.8	10.0	-26.4
Cytidine	0 h at auto-sampler	555.9 ± 27.5	4.9	-
	4 h at auto-sampler	500.4 ± 69.4	13.9	-10.0
	12 h at auto-sampler	521.1 ± 63.5	12.2	-6.3
	24 h at auto-sampler	534.4 ± 63.6	11.9	-3.9
	Storage at -20℃ 1 days	478.3 ± 17.8	3.7	-14.0

	2 Freeze-thaw cycles	474.9 ± 36.7	7.7	-14.6
	Storage at -80°C 15 days	504.8 ± 56.0	11.1	-9.2
	Storage at -80°C 2 months	429.3 ± 41.1	9.6	-22.8
Thymidine	0 h at auto-sampler	45.1 ± 3.1	6.8	-
	4 h at auto-sampler	46.8 ± 5.8	12.4	3.8
	12 h at auto-sampler	47.8 ± 6.0	12.6	6.0
	24 h at auto-sampler	46.4 ± 4.7	10.1	2.8
	Storage at -20°C 1 days	43.2 ± 3.9	9.1	-4.1
	2 Freeze-thaw cycles	39.3 ± 5.3	13.4	-12.8
	Storage at -80°C 15 days	42.4 ± 4.6	10.9	-5.9
	Storage at -80°C 2 months	37.5 ± 2.4	6.4	-16.9
Thymine	0 h at auto-sampler	21.6 ± 1.2	5.4	-
	4 h at auto-sampler	22.9 ± 2.4	10.4	6.1
	12 h at auto-sampler	23.9 ± 1.8	7.6	10.7
	24 h at auto-sampler	20.4 ± 3.1	15.2	-5.2
	Storage at -20°C 1 days	18.8 ± 2.2	11.9	-12.7
	2 Freeze-thaw cycles	19.5 ± 1.5	7.5	-9.6
	Storage at -80°C 15 days	24.2 ± 3.1	12.7	12.3
	Storage at -80°C 2 months	22.1 ± 5.2	23.4	2.5

Table S8. Content determination results of five pyrimidine components (n = 10)

	Uridine (μg/g)	Cytidine (μg/g)	Thymidine (μg/g)	Thymine (μg/g)	Uracil (μg/g)
Control	294.85	3.34	0.39	0.22	58.35
	252.72	4.45	0.41	0.27	49.30
	234.06	3.99	0.39	0.20	43.57
	235.63	2.72	0.32	0.21	48.57
	277.20	3.96	0.54	0.26	67.72
	208.22	2.88	0.36	0.23	58.06
	258.08	2.91	0.24	0.17	40.47
	230.57	2.85	0.41	0.19	43.59
	246.70	3.54	0.78	0.23	49.44
	221.49	2.95	0.27	0.16	37.38
Model	130.78	1.32	0.15	0.14	33.74
	152.74	1.42	0.31	0.22	45.57
	131.23	1.42	0.24	0.21	38.50
	142.41	1.49	0.26	0.13	29.91
	156.85	1.51	0.34	0.20	40.02
	165.96	1.87	0.25	0.23	38.06
	152.90	1.46	0.36	0.25	43.98
	178.65	1.59	0.30	0.24	44.06
	188.51	2.11	0.38	0.19	35.67
	185.54	1.72	0.24	0.20	39.38
INN	155.12	1.4	0.28	0.23	51.83
	173.77	1.58	0.36	0.26	48.40
	219.52	1.72	0.49	0.29	54.63
INN	229.77	1.56	0.37	0.30	60.40
	188.60	1.63	0.38	0.22	52.96

	187.07	1.83	0.37	0.27	51.00
	161.14	1.46	0.34	0.23	48.97
	177.85	1.86	0.29	0.30	56.91
	185.21	1.89	0.35	0.27	59.78
	199.02	1.89	0.47	0.24	43.79
	209.92	2.10	0.40	0.31	68.20
	173.10	1.55	0.41	0.3	49.90
	166.66	1.89	0.44	0.25	54.29
	196.34	1.86	0.86	0.29	53.96
AA-L	204.78	2.17	0.53	0.27	46.84
	176.33	1.71	0.46	0.28	52.03
	256.51	2.22	0.88	0.43	59.37
	184.73	2.18	0.67	0.26	66.21
	220.28	1.97	0.58	0.35	62.01
	227.10	2.10	0.37	0.30	53.29
	175.97	1.86	0.51	0.28	47.06
	180.17	2.04	0.54	0.22	41.82
	201.09	2.28	0.43	0.27	47.86
AA-M	186.43	1.87	0.36	0.35	65.96
	255.71	2.46	0.64	0.26	51.37
	208.93	1.64	0.90	0.48	61.71
	193.72	1.96	0.34	0.34	42.41
	212.80	1.95	0.53	0.29	54.44
AA-M	182.93	1.91	0.43	0.2	47.20
	220.34	2.32	0.43	0.24	40.95
	188.65	1.91	0.26	0.20	45.89
	161.03	1.81	0.34	0.16	35.58
AA-H	186.06	1.68	0.32	0.21	33.16
	207.33	1.49	0.23	0.14	28.28

	208.78	2.47	0.67	0.27	47.42
	129.56	1.82	0.66	0.07	41.36
	161.77	1.86	0.31	0.18	31.29
	157.42	1.70	0.39	0.26	23.85
	153.11	1.32	0.51	0.23	37.98
	222.91	2.44	0.85	0.35	62.73
CAE	252.99	2.80	1.42	0.48	73.07
	246.22	2.30	0.46	0.29	47.99
	239.70	1.83	0.97	0.36	61.13
	196.56	1.64	0.70	0.27	51.09
	221.28	2.48	0.92	0.34	56.42
	219.08	2.22	0.67	0.33	60.03
	210.08	1.59	0.48	0.25	46.04
	253.14	2.40	0.77	0.39	54.91
	308.42	2.80	0.81	0.53	65.49
	206.81	1.82	0.66	0.25	47.87

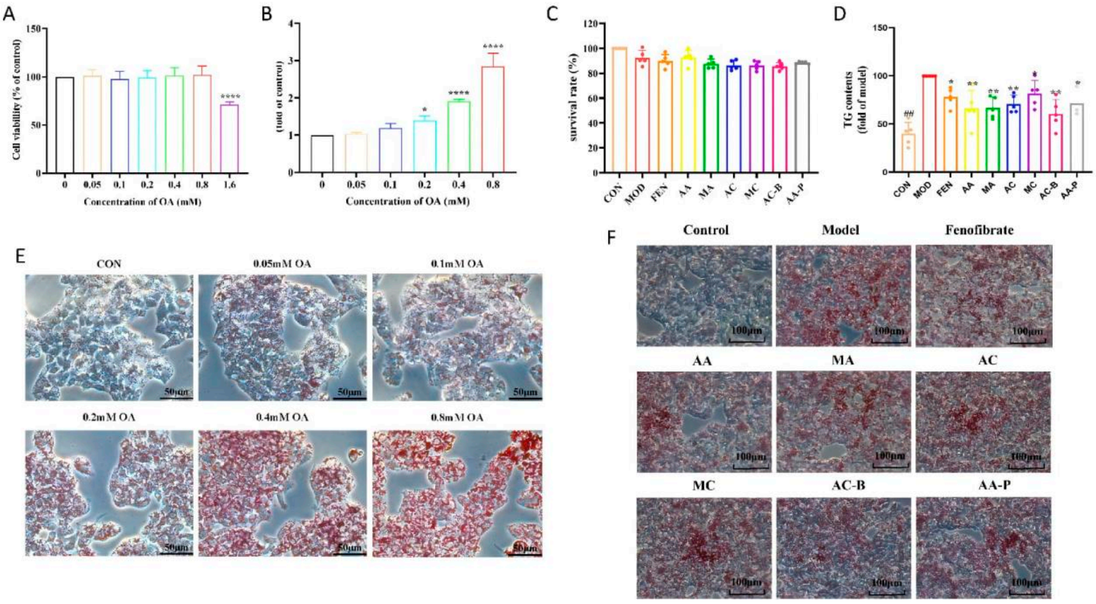

Figure S3. Oleic acid triggers intracellular lipid accumulation in hepatocytes, and the tested compounds alleviate hepatocyte steatosis *in vitro* (n=6).

(A) CCK-8 analysis of cell viability in hepatocytes incubated with gradient concentrations of oleic acid (OA, 0-1.6 mM). Results are normalized to the untreated control group. ****$p < 0.0001$ versus the 0 mM OA group.

(B) Quantitative measurement of intracellular triglyceride (TG) levels in hepatocytes treated with serially diluted OA. *$p < 0.05$, ****$p < 0.0001$ compared with the 0 mM OA control.

(C) Cell survival rate of OA-induced steatotic hepatocytes after treatment with fenofibrate (FEN) and six candidate compounds (AA, MA, AC, MC, AC-B, AA-P). CON, blank control group; MOD, OA-induced steatosis model group.

(D) Relative intracellular TG contents of each group, normalized to the MOD group. $^{##}p < 0.01$ vs. CON group; *$p < 0.05$, **$p < 0.01$ vs. MOD group.

(E) Representative Oil Red O staining micrographs of hepatocytes exposed to increasing OA concentrations (0 - 0.8 mM). Scale bar = 50 μm.

(F) Oil Red O staining for lipid droplets in OA-model hepatocytes supplemented with fenofibrate or target compounds. Scale bar = 100 μm. CON, blank control; MOD, OA steatosis model; FEN, fenofibrate positive control; AA, MA, AC, MC, AC-B, AA-P represent different monomer compounds.

Abbreviations: OA, oleic acid; TG, triglyceride; FEN, fenofibrate.

Table S9. Tab. of primer sequences

Primer Name (Source Species: Human)	Primer Sequence
ACACA primer (forward)	AATAGCGTCTCTAACTTCCTTCAC
ACACA primer (reverse)	CCGTCACTCAGCCGATGTA
Fasn primer (forward)	GGATCACAGGGACAACCTGG
Fasn primer (reverse)	GGGAGATGAGGGGAGTTCCT
Scd1 primer (forward)	GGGGGTGTGCTGACAACTTA
Scd1 primer (reverse)	AGGCCCCTTTTTCTACCAGC
PGM1 primer (forward)	TGGTGAAGATCGTGACAGTTAAG
PGM1 primer (reverse)	GTGGAGATGATACTCTGGATGAAG
UGP2 primer (forward)	AATACTCACCACAGCATCATCAC
UGP2 primer (reverse)	CATTCCTCACACCAATCAGACTT
Lipg primer (forward)	CTCCGTTCCTCTGCTCTGTT
Lipg primer (reverse)	ATACCGCTCATCGTCCATCC
SREBP1 primer (forward)	GATACCACCAGCGTCTACCA
SREBP1 primer (reverse)	TTGCGATGCCTCCAGAAGTA
SREBP2 primer (forward)	GGTTGTCGGGTGTCATGGG
SREBP2 primer (reverse)	TTGCAGCATCTCGTCGATGT
β-actin primer (forward)	AGCGAGCATCCCCCAAAGTT
β-actin primer (reverse)	GGGCACGAAGGCTCATCATT

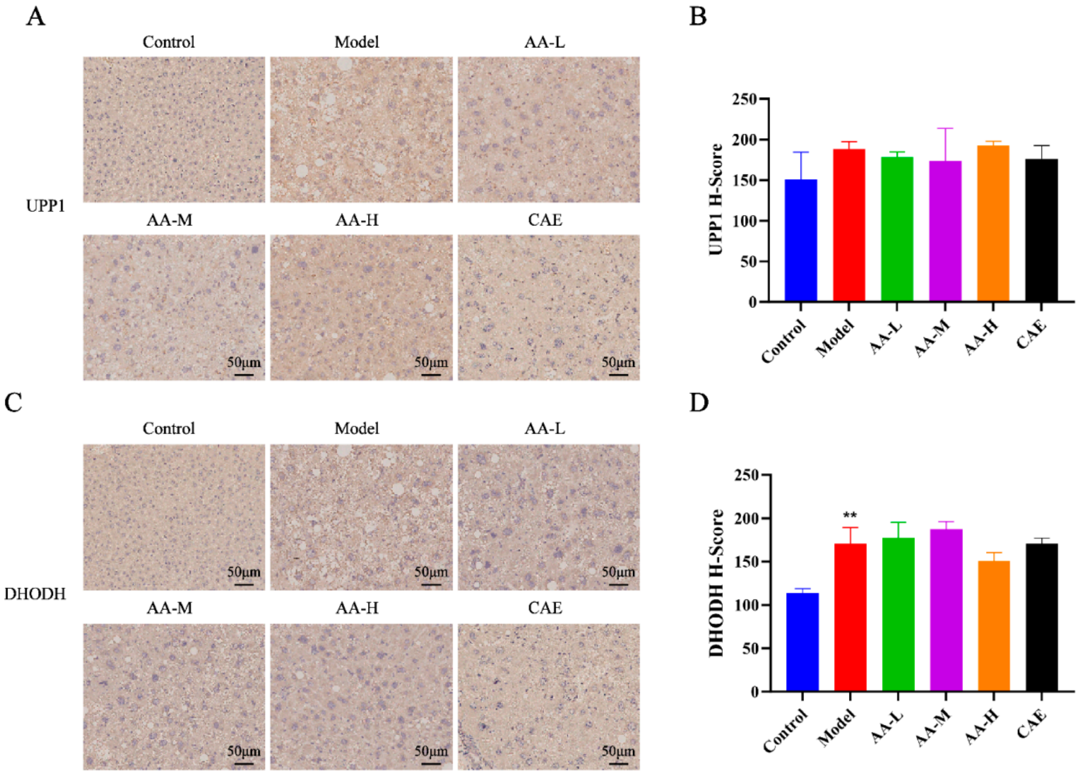

Figure S4. Immunohistochemical analysis of UPP1 and DHODH expression and their quantitative H-Scores in mouse liver. (A) Immunohistochemical staining images of UPP1 in liver tissues (scale bar = 50 μm). (B) Quantitative H-Score analysis of UPP1 expression. (C) Immunohistochemical staining images of DHODH in liver tissues (scale bar = 50 μm). (D) Quantitative H-Score analysis of DHODH expression. Data are presented as mean ± SEM (n=3). **p < 0.01 vs. Control group.

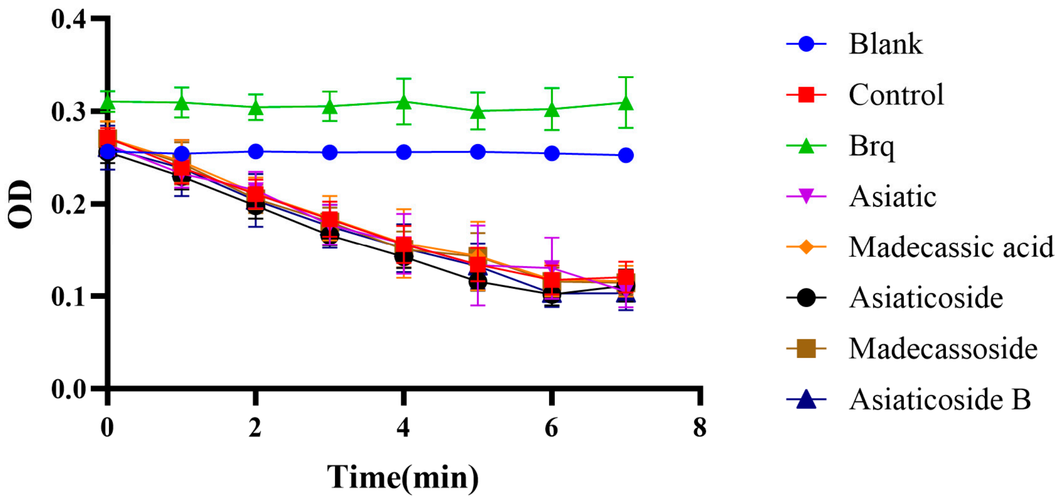

Figure S5. DHODH enzyme kinetic curve ($n = 5$).

Table S10. Docking results of 5 major active components in Centella Asiatica with the UPP1 molecule (kcal/mol)

Target	Compounds				
	Asiatic acid	Madecassic acid	Asiaticoside	Madecassoside	Asiaticoside B
UPP1	-9.3	-7.4	-8.5	-8.6	-9.2
